# Single-dose genome editing therapy rescues auditory and vestibular functions in adult mice with DFNA41 deafness

**DOI:** 10.1172/JCI187872

**Published:** 2025-08-14

**Authors:** Wei Wei, Wenliang Zhu, Stewart Silver, Ariel M. Armstrong, Fletcher S. Robbins, Arun Prabhu Rameshbabu, Katherina Walz, Yizhou Quan, Wan Du, Yehree Kim, Artur A. Indzhykulian, Yilai Shu, Xue-Zhong Liu, Zheng-Yi Chen

**Affiliations:** 1 Department of Otolaryngology-Head and Neck Surgery, Graduate Program in Speech and Hearing Bioscience and Technology and Program in Neuroscience, Harvard Medical School, Boston, Massachusetts, USA.; 2 Eaton-Peabody Laboratory, Massachusetts Eye and Ear, Boston, Massachusetts, USA.; 3 Dr. John T. Macdonald Foundation Department of Human Genetics and; 4 John P. Hussman Institute for Human Genomics, University of Miami Miller School of Medicine, Miami, Florida, USA.; 5 ENT Institute and Otorhinolaryngology Department of Eye & ENT Hospital, State Key Laboratory of Medical Neurobiology and MOE Frontiers Center for Brain Science,; 6 Institutes of Biomedical Science, and; 7 NHC Key Laboratory of Hearing Medicine, Fudan University, Shanghai, China.; 8 Department of Otolaryngology, University of Miami Miller School of Medicine, Miami, Florida, USA.

**Keywords:** Otology, Therapeutics, Gene therapy, Genetic diseases, Mouse models

## Abstract

Genome editing has the potential to treat genetic hearing loss. However, current editing therapies for genetic hearing loss have shown efficacy only in hearing rescue. In this study, we evaluated a rescue strategy using adeno-associated virus (AAV) type 2–mediated delivery of *Staphylococcus aureus* Cas9-sgRNA in the mature inner ear of the *P2rx2^V61L/+^* mouse model of autosomal dominant deafness-41 (DFNA41), a dominant, delayed-onset, and progressive hearing loss in humans. We demonstrate that local injection in adult mice results in efficient and specific editing that abolishes the mutation without notable off-target effects or AAV genome integration. Editing effectively restores long-term auditory and vestibular function. Editing further protects *P2rx2^V61L/+^* mice from hypersensitivity to noise-induced hearing loss, a phenotype also observed in patients with DFNA41. Intervention in mice at a juvenile stage broadens the frequency range rescued, highlighting the importance of early intervention. An effective and specific gRNA for the human *P2RX2* V60L mutation has been identified. Our study establishes the feasibility of editing to treat DFNA41 caused by *P2RX2* V60L mutation in humans and opens an avenue for using editing to rescue hearing and vestibular function while mitigating noise-induced hearing loss.

## Abstract 2

<strong>Gene editing rescues hearing and vestibular function in adult *P2rx2^V61L/+^* mouse model of deafness DFNA41</strong>

## Introduction

Congenital hearing loss is the most prevalent birth defect worldwide (1). In developed countries, genetic factors account for congenital sensorineural hearing loss in as many as 1 in 500 newborns (2). Despite the identification of more than 150 deafness genes, effective biological treatments for preserving or reversing hereditary hearing loss remain elusive (3). The current clinical options for sensorineural hearing loss are limited to hearing aids and cochlear implants, which have substantial limitations, including poor speech recognition in noisy environments (3, 4) and unsatisfactory music perception, partly due to incomplete synchrony restoration and device inconvenience (3, 5, 6). These limitations highlight the urgent need for novel therapeutic approaches.

Gene therapy, including editing therapy, has emerged as a promising strategy for treating genetic hearing loss. Remarkable progress has been made in developing gene therapy strategies for genetic hearing loss over the past decades, encompassing gene replacement (7–9), gene augmentation (10–13), gene silencing (14–16), and genome editing (17–22). Despite the remarkable success of gene therapy in treating genetic hearing loss in mouse models, the majority of studies, including our previous genome editing studies (20, 22), have been conducted in the neonatal stage (7, 20, 23–33). In neonatal mice, the inner ear is still developing (34, 35), and the treatment effect likely resembles that achievable in the human fetal inner ear. In humans, however, newborn inner ears are fully mature (36, 37). To successfully treat genetic hearing loss in humans, demonstrating efficacy in the fully mature mouse inner ear is a prerequisite. Currently, successful adult intervention has only been achieved in *Otof*, *Tmprss3*, and *Mir96* mouse models (13, 38, 39), and no other gene replacement or editing therapies have succeeded via adult intervention. This likely is due, in part, to severe degeneration of relevant cell types or insufficient adult cell targeting.

In this study, we aimed to address the challenges of treating delayed-onset, progressive, dominant hearing loss and vestibular dysfunction in the autosomal dominant deafness-41 (DFNA41) mouse model by demonstrating successful rescue through a single adult-stage injection using precise genome editing. We used a *P2rx2^V61L/+^* knock-in mouse model that faithfully reproduced the phenotypes of patients with DFNA41 carrying a dominant *P2RX2* V60L gain-of-function mutation (40, 41). The *P2RX2* V60L mutation has been reported to specifically reduce the ATPase activity of the P2RX2 protein and impair activation of the P2RX2 channel (42). Our approach involved the use of a genomic editing complex, delivered by adeno-associated virus type 2 (AAV2), designed to specifically disrupt the mutant allele while preserving the function of the WT allele for hearing rescue. The AAV-editing complex was delivered locally via round window membrane injection with canal fenestration (RWM+CF) in adult DFNA41 mice, resulting in a highly effective and sustained preservation of auditory function, along with the rescue of vestibular function. Mutation-specific editing further rescued DFNA41 mice from increased susceptibility to noise-induced hearing loss (NIHL).

Our editing approach showed a robust safety profile, including minimal off-target effects and the absence of AAV integration in the genomic DNA. More efficacious outcomes in juvenile DFNA41 mice underscore the importance of early intervention in patients with DFNA41. Last, we have identified an efficient and specific gRNA targeting the human DFNA41 V60L mutation, paving the way for clinical translation application.

## Results

### Screening of genome editors for P2rx2^V61L^ allele-specific editing.

**Figure 1:**
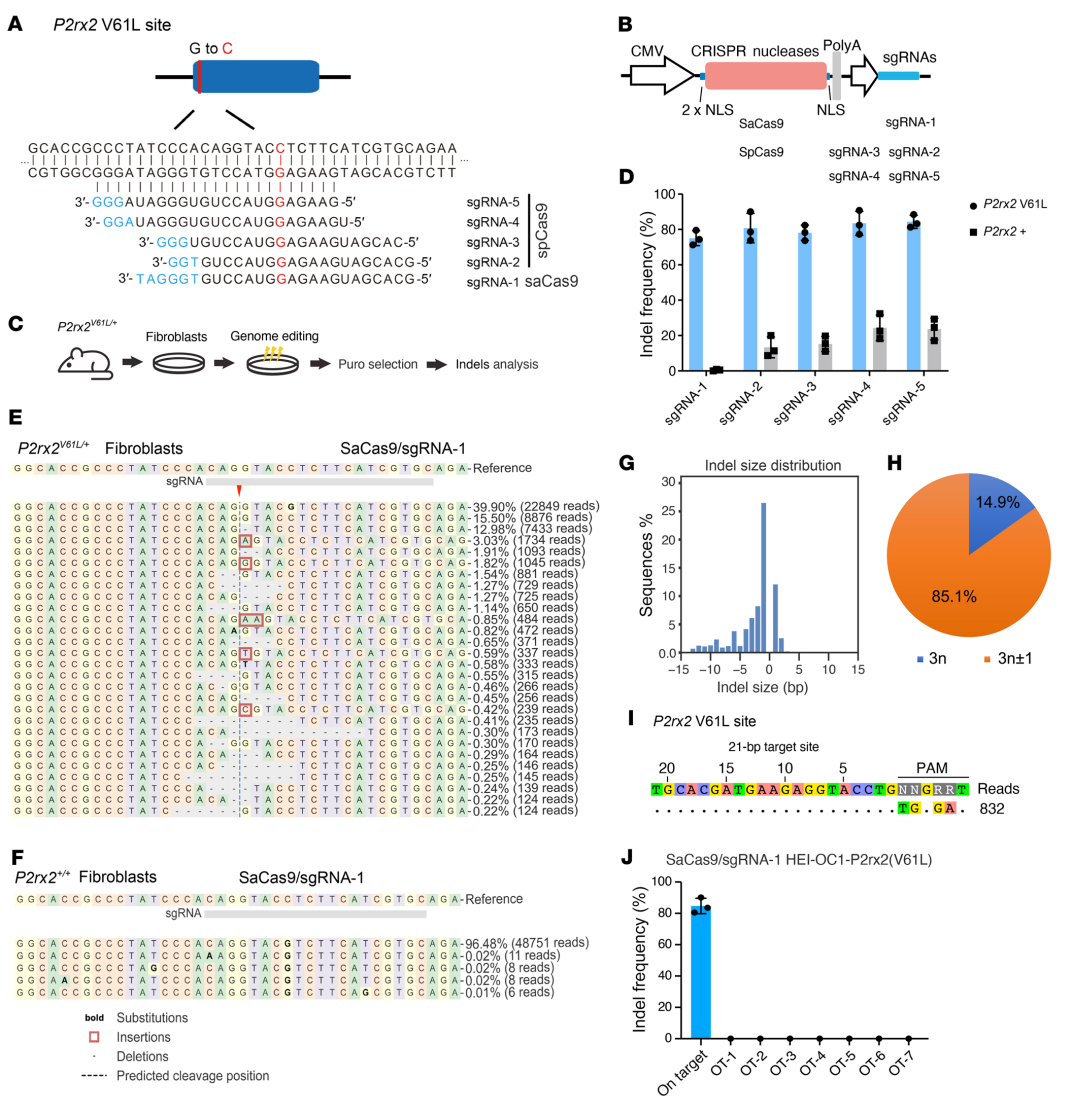
Allele-specific genome editing using SaCas9–sgRNA-1 in mouse *P2rx2^V61L/+^* primary cells. (**A**) The DNA sequence of the *P2rx2^V61L^* mutation locus and the sgRNAs designs for SpCas9 and SaCas9, respectively. The mutation nucleotide (**C**) in *the P2rx2^V61L^* allele is in red. The protospacer adjacent motifs (PAMs) nucleotides are in blue. (**B**) Schematic overview of plasmid constructions for different CRISPR systems used for the in vitro study. (**C**) The experiment design for studying genome editing in the primary fibroblasts from *P2rx2^V61L/+^* mice. (**D**) Quantification of the indel frequency in *P2rx2^V61L/+^* and *P2rx2^+/+^* primary fibroblasts after genome editing using different Cas9/sgRNA combinations. *n* = 3. Error bar represents SD. (**E** and **F**) Representative NGS results from SaCas9–sgRNA-1–edited *P2rx2^V61L/+^* (**E**) and *P2rx2^+/+^* primary fibroblasts (**F**). The red arrowhead indicates the double-stranded DNA cutting site. (**G**) Indel profiles from the SaCas9–sgRNA-1–edited *P2rx2^V61L^* allele in primary fibroblasts. Negative numbers represent deletions, positive numbers represent insertions. (**H**) Pie chart showing the out-of-frame (3n ± 1) ratio in the indel profile. (**I**) CIRCLE-Seq analysis of SaCas9–sgRNA-1 in *P2rx2^V61L/+^* primary fibroblasts’ genomic DNA. (**J**) Quantification of indel frequency of potential off-target sites from the mouse genome. NLS, nuclear localization signal. Puro, puromycin.

To identify the most effective genome editor for targeting the *P2rx2^V61L^* allele, we designed sgRNAs for use with *Staphylococcus aureus* Cas9 (SaCas9) sgRNA1 and *Streptococcus pyogenes* Cas9 (SpCas9) sgRNA2–sgRNA5 nucleases (Figure 1A). All Cas9/sgRNA combinations were incorporated into the same plasmid backbone containing 3 nuclear localization signals and optimized sgRNA scaffold (43) (Figure 1B). The Cas9/sgRNA plasmids and a puromycin resistance plasmid were cotransfected into the primary fibroblasts derived from *P2rx2^V61L/+^* mice, using nucleofection. The puromycin-resistant cells were collected for DNA extraction, amplicon amplification, and analysis by next-generation sequencing (NGS) for insertion-deletion (indel) identification (Figure 1C).

In primary fibroblasts, efficient editing, as indicated by indel reads, was detected in the *P2rx2^V61L^* allele across all gRNA designs for SpCas9 and SaCas9, with editing efficiencies ranging from 71.28% to 83.29% (Figure 1D). For SpCas9 sgRNAs (sgRNA-2, -3, -4 and -5), elevated levels of indels were detected in WT *P2rx2^+^* allele (8.73%–29.20%) (Figure 1D, and Supplemental Figure 1, A and B; supplemental material available online with this article; https://doi.org/10.1172/JCI187872DS1), indicating undesired off-target editing on the WT allele. The findings suggested that SpCas9/sgRNAs can tolerate 1 nucleotide mismatch between the *P2rx2^V61L^* allele and the WT allele, making it unsuitable for allele-specific genome editing. In contrast, NGS analysis of SaCas9–sgRNA-1 revealed a high level of indel formation in the *P2rx2^V61L^* allele (75.01% ± 4.55%) (Figure 1, D and E), with negligible indels observed in the WT allele (0.45 ± 0.39%) (Figure 1, D and F), indicating *P2rx2^V61L^* allele-specific editing. For SaCas9–sgRNA-1–mediated editing, NGS revealed a variety of indels, with single-nucleotide deletions being the most common type (Figure 1G). Most of the indels (85.1%) caused frameshift mutations (Figure 1H), suggesting efficient disruption of the P2rx2*^V61L^* protein by SaCas9–sgRNA-1.

To assess the editing efficiency and specificity in the cochlear cells, we used the mouse organ of Corti cell line HEI-OC1 to generate HEI-OC1-P2rx2-V61L cells (OC1-V61L), which harbor the *P2rx2^V61L^* DNA fragment in the genome using the PiggyBac system (Supplemental Figure 1C). NGS analysis of HEI-OC1-V61L cells after transfection with SaCas9–sgRNA-1 or SpCas9/sgRNA-2 plasmids revealed similarly robust indel formation. In the HEI-OC1 cells harboring the WT allele, SpCas9/sgRNA-2–mediated editing was evident from indel analysis, consistent with the primary fibroblasts results (Supplemental Figure 1D and Figure 1D). In contrast, SaCas9–sgRNA-1 transfection produced negligible editing events in the HEI-OC1 cells with the WT allele (Supplemental Figure 1, D and G). The indel profile in SaCas9–sgRNA-1–edited HEI-OC1-V61L cells showed various types of indels (Supplemental Figure 1, E and F), with single-nucleotide deletions being the most common.

To comprehensively assess off-target editing, we performed circularization for in vitro reporting of cleavage effects by sequencing (CIRCLE-Seq) (44) on HEI-OC1-V61L cells edited by SaCas9–sgRNA-1 and identified no off-target sites beyond the on-target locus (Figure 1, I and J). Furthermore, computational predictions were used to identify potential off-target loci (45, 46). Analysis of the top 7 predicted off-target sites, identified using the cutting frequency determination score (45) in SaCas9–sgRNA-1–edited HEI-OC1-V61L cells, revealed no off-target editing events, whereas on-target editing efficiency exceeded 80% (Figure 1D and Supplemental Figure 1H). Collectively, these results demonstrate that SaCas9–sgRNA-1 specifically targets the *P2rx2^V61L^* allele with minimal off-target effects in mouse primary fibroblasts or a cochlear cell line.

### In vivo genome editing of the P2rx2^V61L^ allele in the cochlea of adult mice.

**Figure 2:**
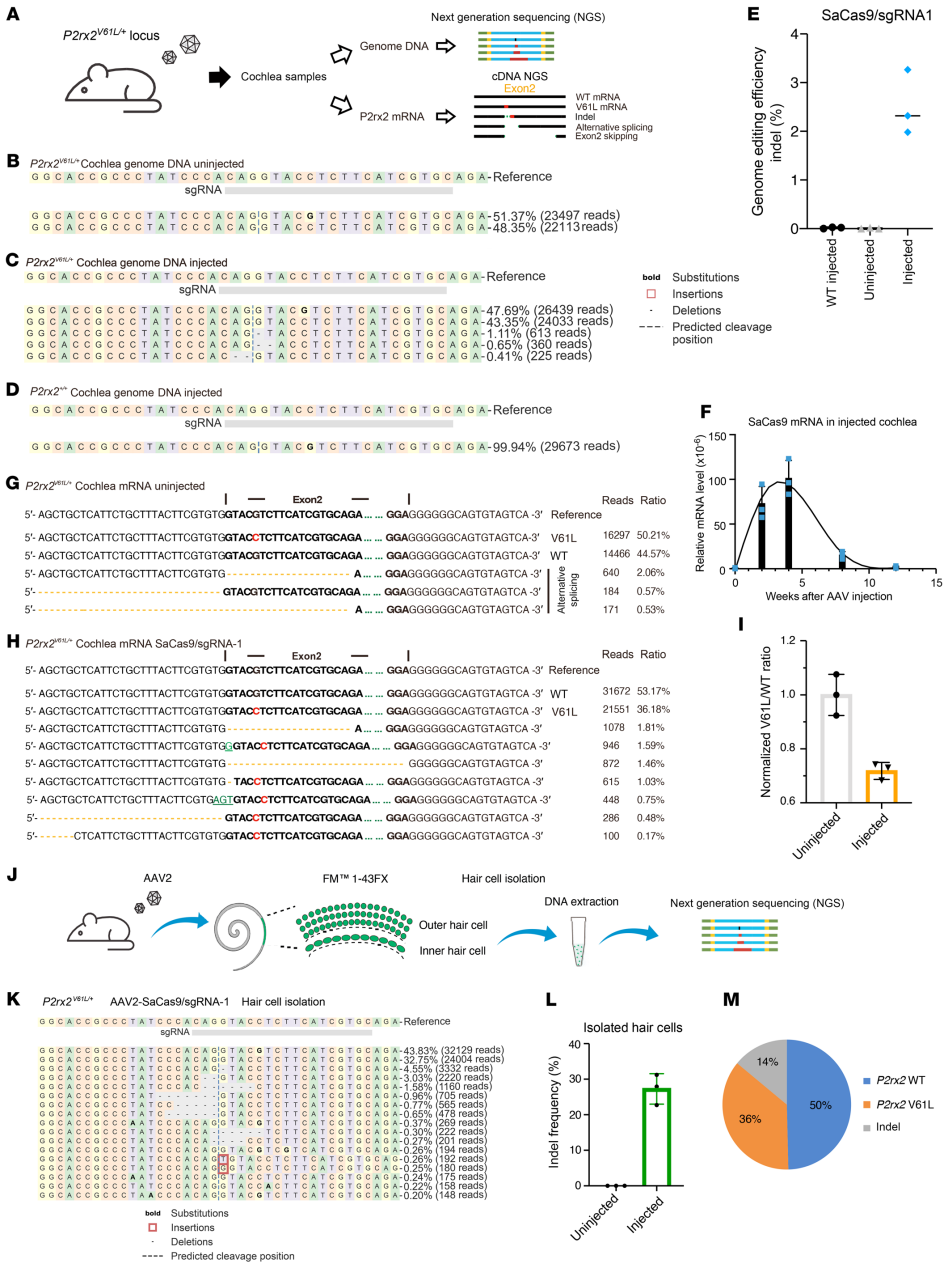
AAV2-mediated genome editing at the *P2rx2* V61L locus in the cochlea of adult *P2rx2^V61L/+^* mice. (**A**) Experimental overview for in vivo studies. (**B**–**D**) Representative NGS showed indels from uninjected (**B**), injected (**C**) *P2rx2^V61L/+^*, and injected WT (*P2rx2^+/+^*) mice (**D**). (**E**) Quantification of indel frequency of NGS results from injected and uninjected *P2rx2^V61L/+^* ears and injected *P2rx2^+/+^* ears (*n* = 6 for each). Cochleae were collected 8 weeks after AAV injection. Each dot represents a unique sequencing reaction from 2 cochlea combined. Error bar represents SD. (**F**) qPCR analysis of SaCas9 mRNA level in the injected cochlea at different time points after injections (*n* = 6). Each dot represents an independent result from 2 cochleae combined. Values and error bars reflect mean ± SD. (**G**) NGS reads showed the distribution of *P2rx2^+^* and mutant *P2rx2^V61L^* transcripts from uninjected *P2rx2^V61L/+^* mice. (**H**) NGS reads showed decreased *P2rx2^V61L^* transcripts compared with WT *P2rx2* transcripts from AAV2–SaCas9–sgRNA-1 injected *P2rx2^V61L/+^* ears. (**I**) A normalized ratio of unmodified *P2rx2^V61L^* transcripts relative to unmodified WT transcripts in uninjected and injected *P2rx2^V61L/+^* animals based on the NGS reads. *n* = 3. (**J**) Schematic overview of the experimental protocol of HC isolation, cell lysis, and NGS. (**K**) Representative NGS result of isolated HC DNA from AAV2–SaCas9–sgRNA-1–injected *P2rx2^V61L/+^* cochleae. (**L**) Quantification of *P2rx2^V61L^* allele-specific indel frequency of the NGS results from injected and uninjected *P2rx2^V61L/+^* HC lysis (*n* = 3). The error bar represents SD. (**M**) The distribution of WT allele reads, *P2rx2^V61L^* allele reads, and indel-containing reads of the NGS results from AAV2–SaCas9–sgRNA-1–injected *P2rx2^V61L/+^* HCs.

To evaluate the therapeutic potential of SaCas9–sgRNA-1, we packaged it in AAV2 and administered it to the cochlea of 4-week-old *P2rx2^V61L/+^* mice via RWM+CF at a dose of 4 × 10^9^ vg/ear. After 8 weeks, the cochleae were harvested, and DNA and RNA were extracted for NGS and indel analysis (Figure 2A). In uninjected mice, background indel frequencies ranged between 0% and 0.05%. In vivo NGS of the cochlea showed the presence of indels at the *P2rx2^V61L^* locus in the injected *P2rx2^V61L/+^* ears after 8 weeks, with an indel rate of 2.62% ± 0.64% and no detectable editing of the WT allele (Figure 2, B and C). We also injected AAV2–SaCas9–sgRNA-1 into the cochlea of WT adult mice; NGS analysis did not detect indels in the injected WT cochlea (Figure 2, D and E). We determined the expression of SaCas9 after cochlear injection by qPCR. SaCas9 mRNA level peaked around 4 weeks after injection, followed by a decline over time (Figure 2F). By the 12th week after injection, SaCas9 expression was no longer detectable (Figure 2F), which potentially is attributable to either CMV promoter silencing or the immune response triggered by SaCas9 in adult mice, as reported by previous studies (43, 47–49).

Given that on-target editing efficiency would directly impact hearing rescue, we measured editing efficiency by examining changes in *P2rx2* transcripts in the injected *P2rx2^V61L/+^* cochleae. *P2rx2* is specifically expressed in the hair cells (HCs). We performed NGS analysis of cDNAs from injected and uninjected contralateral control cochleae and detected multiple indel-containing *P2rx2* transcripts in the injected samples, whereas none were found in the uninjected control cochleae (Figure 2, G and H). Additionally, exon 2–skipping *P2rx2* transcripts were detected in the injected cochleae, accounting for 1.46% of total reads, suggesting that genome editing at the *P2rx2^V61L^* locus disrupted normal splicing (Figure 2H). This is likely due to the SaCas9–sgRNA-1 cutting site targeting the exon 2 splicing acceptor, which was disrupted by indels. Rare, alternatively spliced transcripts were detected in both uninjected and injected samples, constituting less than 5% of all reads (Figure 2, G and H). This suggests that genome editing did not alter the expression of these rare transcripts.

Compared with uninjected *P2rx2^V61L/+^* cochleae, a significant decrease in the ratio of unedited *P2rx2^V61L^* transcripts to WT *P2rx2* transcripts was observed in the injected ears (Figure 2I). After SaCas9–sgRNA-1–mediated editing of the *P2rx2^V61L^* allele, the proportion of unedited *P2rx2^V61L^* transcripts in the injected ears decreased by 28.2%, to 71.2% ± 3.0%, relative to the uninjected ears (Figure 2I). These findings suggest that approximately 28% of *P2rx2*-expressing cells were successfully edited at the *P2rx2^V61L^* allele through genome editing. Given that cells in *P2rx2^V61L/+^* cochleae carry only 1 copy of the V61L allele, these findings suggest that genome editing successfully disrupted the mutant allele in approximately 28% of *P2rx2*-expressing HCs.

To precisely determine editing efficiency in HCs, we isolated HCs using the FM1-43 uptake assay (Supplemental Figure 2A). Eight weeks after injection of SaCas9–sgRNA-1 into adult *P2rx2^V61L/+^* ears, the cochleae were dissected and the cells dissociated using 0.25% trypsin-EDTA, then incubated with FM1-43FX to label HCs through open mechanotransduction channels (50, 51). Subsequently, the labeled HCs were collected and lysed to obtain genomic DNA, which was then analyzed for indel frequency by NGS (Figure 2J). In contrast to uninjected cochleae, a wider range of indel types was observed in isolated HCs (Figure 2K), with an indel frequency of 26.96% ± 4.2% in the *P2rx2^V61L^* allele (Figure 2L). The editing frequency based on the HC DNA was consistent with that from the *P2rx2^V61L^* transcript analysis. Furthermore, the combined reads from the edited and unedited *P2rx2^V61L^* locus were equal to the *P2rx2^+^* reads in the injected ear (Figure 2M). This finding indicates that no noticeable chromatin rearrangements (e.g., lesions, large deletions, insertions) were caused by genome editing, reinforcing the safety and accuracy of the approach in targeted HCs.

AAV integration into the genome as a consequence of induced DNA breaks (48, 52) is a potential safety concern in editing therapy via AAV delivery (Supplemental Figure 2B). To study AAV integration at the target locus in the inner ear, we designed specific primers for PCR and NGS to detect integration between the *P2rx2* locus and AAV inverted terminal repeats (ITRs) (Supplemental Figure 2C). We first evaluated AAV integration in the HEK-293T-P2rx2-V61L cells after AAV2–SaCas9–sgRNA-1 infection. A low level of *P2rx2^V61L^*-ITR integration was observed, and the rate of integration was correlated with AAV2–SaCas9–sgRNA-1 dose (Supplemental Figure 2D). At a dose of 10^3^ vg/cell, on-target editing approached peak efficiency with virtually no detectable integration (Supplemental Figure 2D). At the AAV dose exceeding 10^4^ vg/cell, a *P2rx2^V61L^*-ITR integration rate of 5.0% ± 0.2% was detected (Supplemental Figure 2D). These findings strongly support that optimizing AAV dose allows for maximum editing efficiency while minimizing AAV integration.

We further confirmed that no *P2rx2^V61L^*-ITR integration reads were detected in isolated HCs from in vivo samples after AAV2–SaCas9–sgRNA-1 injection (Supplemental Figure 2, E and F). Furthermore, qPCR analysis of the *P2rx2^V61L^*-ITR transcript revealed no significant difference between uninjected and injected animals (Supplemental Figure 2G), indicating the absence of *P2rx2^V61L^*-ITR chimeric transcripts caused by genome editing. Together, these results demonstrate that AAV2-mediated genome editing at the *P2rx2^V61L^* locus exhibits negligible AAV integration in the injected mature cochlea, providing a crucial piece of evidence for the safety and specificity of the therapeutic approach.

### AAV2 transduces cochlear HCs in adult mice.

AAV2 is a well-established AAV serotype known for its high transduction efficiency in inner hair cells (IHCs) and outer hair cells (OHCs) of the cochlea, with preferential distribution from the apical turn to the basal turn (13). Given its effective transduction capabilities, we selected AAV2 as the vector for genome editing therapy in *P2rx2^V61L/+^* mice.

To assess its distribution and transduction efficiency, we injected 1 × 10^10^ vg of AAV2-GFP with a titer of 1 × 10^13^ vg/mL into the cochleae of 4-week-old mice via RWM+CF (Supplemental Figure 3A). Cochleae were harvested 4 weeks after injection and analyzed for GFP expression across the apical (8 kHz), middle (16 kHz), and basal (32 kHz) turns (Supplemental Figure 3, B and C). AAV2-GFP efficiently transduced all IHCs across all the cochlear turns (Supplemental Figure 3D). In OHCs, transduction efficiency varied, decreasing from the apex to the base (Supplemental Figure 3D). Intensity quantification revealed uniformly high GFP intensity in IHCs, whereas intensity in OHCs was reduced to approximately 50% in the apex and approximately 25% in the base compared with IHCs (Supplemental Figure 3E).

Because the titer of AAV2–SaCas9–sgRNA was 4.46 × 10^12^ vg/mL, we injected 4 × 10^9^ vg of AAV2-GFP into the cochleae of 4-week-old mice to better match the viral dose (Supplemental Figure 3F). Four weeks after the injection, we observed that IHC transduction remained at 100%, whereas OHC transduction rates were approximately 80% in apical and middle turns and 65% in the basal turn (Supplemental Figure 3, G and H). The auditory brainstem response (ABR) and distortion product otoacoustic emissions (DPOAE) tests showed no hearing loss in injected ears compared with uninjected controls (Supplemental Figure 3, I and J).

In summary, AAV2 effectively transduces 100% of IHCs with robust transgenes expression. However, it transduces OHCs less efficiently, both in transduction rate and expression level. AAV2 administration through RWM + CF does not impair normal hearing in adult mice.

### AAV2–SaCas9–sgRNA-1 delivery in adult mice promotes P2rx2^V61L/+^ HC survival, restores IHC length, and preserves HC morphology.

**Figure 3:**
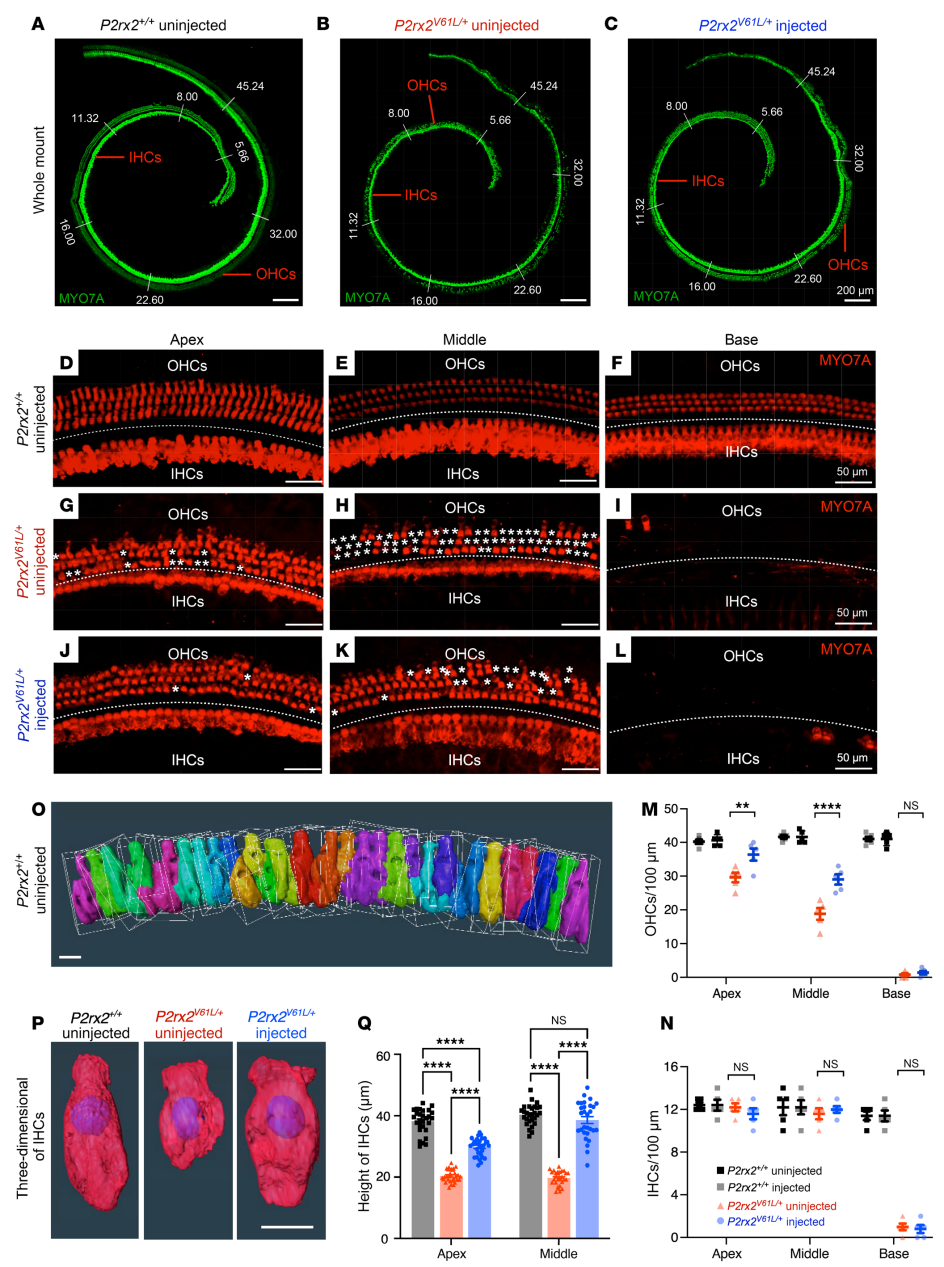
AAV2–SaCas9–sgRNA-1 adult injection rescues OHC and IHC length in *P2rx2^V61L/+^* mouse model of DFNA41. (**A**–**C**) Representative confocal microscopy images of whole mounts of intact MYO7A-labeled (green) cochleae of WT (**A**), uninjected (**B**), and injected (**C**) *P2rx2^V61L/+^* mice, 9 months after 4-week injection. Scale bars: 200 μm. (**D**–**L**) Representative confocal microscopy images of the surface views of WT, uninjected, and injected *P2rx2^V61L/+^* cochleae at the apex (**D**, **G**, and **J**), middle (**E**, **H**, and **K**) and base (**F**, **I**, and **L**), respectively. The asterisks indicate OHC loss in the apical and the middle turns, and severe IHC loss was seen in the base turn of uninjected and injected *P2rx2^V61L/+^* cochleae. Scale bars: 50 μm. (**M**) Quantification of OHCs at the apex, middle, and base turns of the cochleae from WT, uninjected, and injected *P2rx2^V61L/+^* ears. (**N**) Quantification of IHCs at the apex, middle, and base turns of the cochleae among WT, uninjected, and injected *P2rx2^V61L/+^* ears. (**O**) Schematic representation of 3D surface projection of IHCs in the *P2rx2^+/+^* uninjected group from **D** using Amira bounding box analysis. (**P**) Representative 3D surface projection of IHCs from **D**, **G**, and **J** in *P2rx2^+/+^* uninjected, *P2rx2^V61L/+^* uninjected, and *P2rx2* ^V61L^ *^/+^* injected ears. Scale bar: 10 μm. (**Q**) Quantification of IHC length in the apex and the middle turns. Data are presented as mean ± SEM. Two-way ANOVA determined significance with Bonferroni correction for multiple comparisons. **P < 0.01, ****P < 0.0001.

To evaluate the impact of AAV2–SaCas9–sgRNA-1–mediated CRISPR/Cas9 editing on HC survival, we examined HC numbers at 9 months after injection at 4 weeks of age in *P2rx2^V61L/+^* mice. Compared with WT inner ears, uninjected *P2rx2^V61L/+^* ears had significant loss of HCs across all cochlear turns, as shown by immunolabeling and quantification, with complete loss in the basal turn and a partial loss in the middle and apical turns (Figure 3, A, B, D–I, M, and N). In the injected *P2rx2^V61L/+^* ears, a complete loss of basal HCs was observed (Figure 3, C, L, M, and N). However, significantly more HCs, especially OHCs, survived in the middle and apical turns (Figure 3, C, J, K, M, and N), compared with the uninjected *P2rx2^V61L/+^* ears (Figure 3, B, G, H, M, and N). We conclude that AAV2–SaCas9–sgRNA-1–mediated editing markedly promotes long-term HC survival, especially in the middle and apical turns.

Mature IHCs have an elongated shape with a length of approximately 27 μm at 1 month and 31 μm at 4 months of age (41). In contrast, we found that IHC length was shortened to approximately 21 μm between 1 and 4 months of age in adult *P2rx2^V61L/+^* mice (41). To assess the effect of AAV2–SaCas9–sgRNA-1 on IHC length, we performed 3D reconstruction and analysis (Figure 3O). Nine months after injection at 4 weeks of age, IHC lengths in injected ears were approximately 9 μm longer in the apical turn and 18 μm longer in the middle turn compared with uninjected ears (Figure 3, P and Q). These results suggest AAV2–SaCas9–sgRNA-1 significantly ameliorates IHC length abnormalities in adult *P2rx2^V61L/+^* mice.

**Figure 4:**
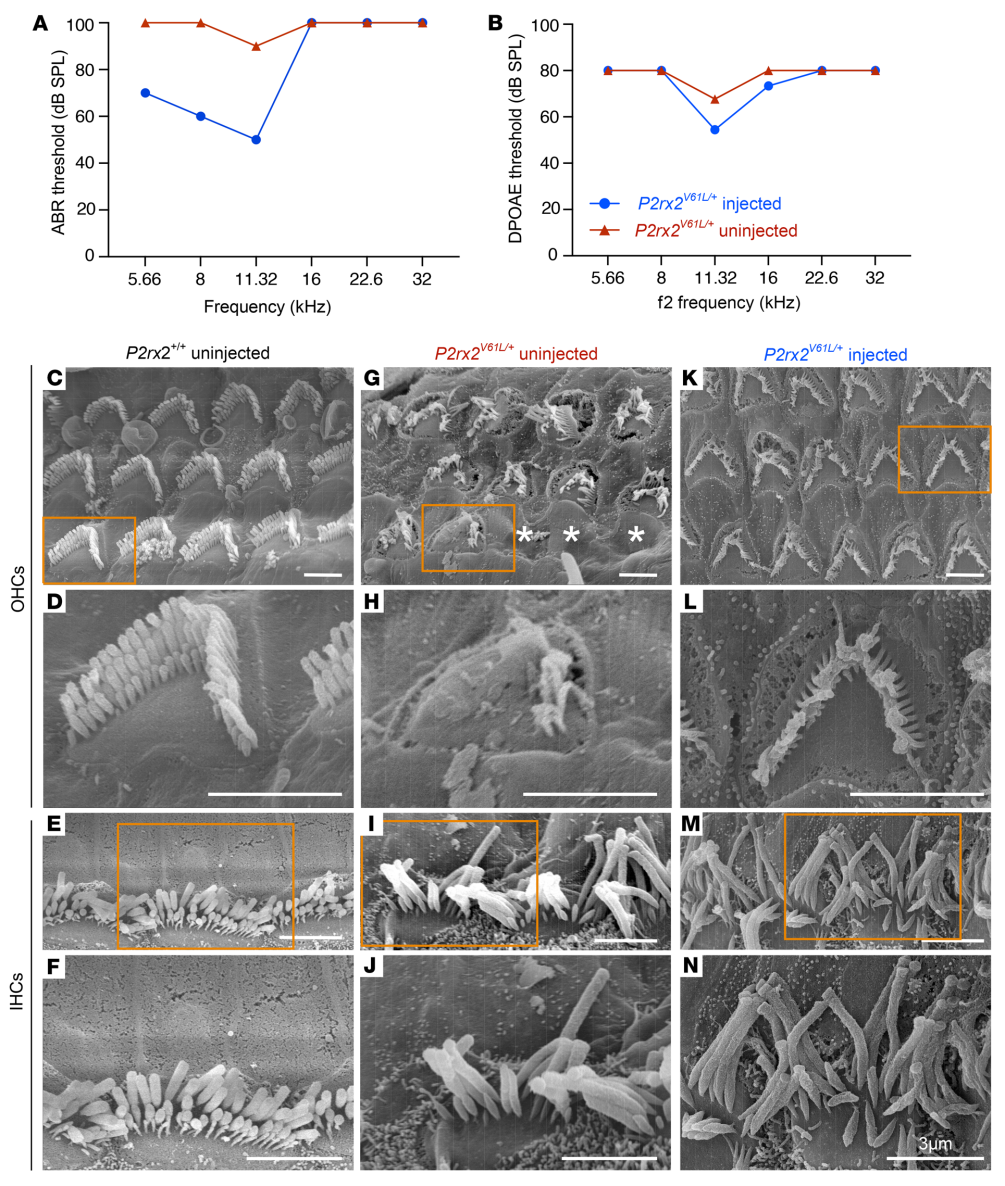
AAV2–SaCas9–sgRNA-1 rescues HC morphology in the *P2rx2^V61L/+^* mouse model of DFNA41. (**A** and **B**) Representative ABR (**A**) and DPOAE (**B**) thresholds of injected and uninjected contralateral ears of a *P2rx2^V61L/+^* mouse 9 months after injection. (**C**–**F**) Representative HC stereocilia morphology analyzed by scanning electron microscope at the apical turn of *P2rx2^+/+^* cochlear OHC (**D**, [an enlarged inset from **C**]) and IHC (**F** [an enlarged inset from **E**]). (**G**–**J**) Representative scanning electron microscopy images of the apical turn of uninjected *P2rx2^V61L/+^* stereocilia of OHC (**H** [an enlarged inset from **G**]), and IHC (**J** [an enlarged inset from **I**]). Asterisks represent missing OHCs (**G**). (**K**–**N**) Representative scanning electron microscopy images of the apical turn of AAV2–SaCas9–sgRNA-1–injected *P2rx2^V61L/+^* stereocilia of OHCs (**L** [an enlarged inset from **K**]), and IHC (**N**, [an enlarged inset from **M**]. Scale bars: 3 μm.

The structure of HC stereocilia is critical for function and hearing, with P2X2 receptors most abundant on the stereocilia and cuticular plates facing the endolymph (53, 54). To evaluate the effect of AAV2–SaCas9–sgRNA-1 on the stereocilia structure, we performed scanning electron microscopy to examine the apex-middle turn HC stereocilia of WT, treated, and untreated *P2rx2^V61L/+^* ears at 9 months after injection at 4 weeks of age. Hearing was rescued at 3 frequencies in the injected ears (Figure 4, A and B). In WT ears, the stereocilia of OHC and IHC were well organized (Figure 4, C–F). In contrast, uninjected P2rx2^V61L/+^ ears exhibited absent, partially missing, or disorganized OHC stereocilia (Figure 4, G and H). The stereocilia of IHCs were present but lacked the shorter stereocilia (Figure 4, I and J). In injected ears, the overall V-shape pattern of OHC stereocilia, as well as the structure, was maintained (Figure 4, K and L). The stereocilia of IHC were present in the injected ears but were generally longer (Figure 4, M and N). These scanning electron microscopy data reveal that AAV2–SaCas9–sgRNA-1 ameliorates HC stereocilia defects, especially in OHCs of *P2rx2^V61L/+^* mice, supporting its potential to preserve HC morphology and function.

### Adult genome editing therapy preserves auditory function in P2rx2^V61L/+^ mice.

The *P2rx2^V61L/+^* mouse model had delayed-onset hearing loss at P21 and progressed to profound deafness over time (41). This pattern mirrors the onset of hearing loss in patients with DFNA41, which typically is detected in the second decade of life (55), suggesting that P21 could be an effective intervention time point.

**Figure 5:**
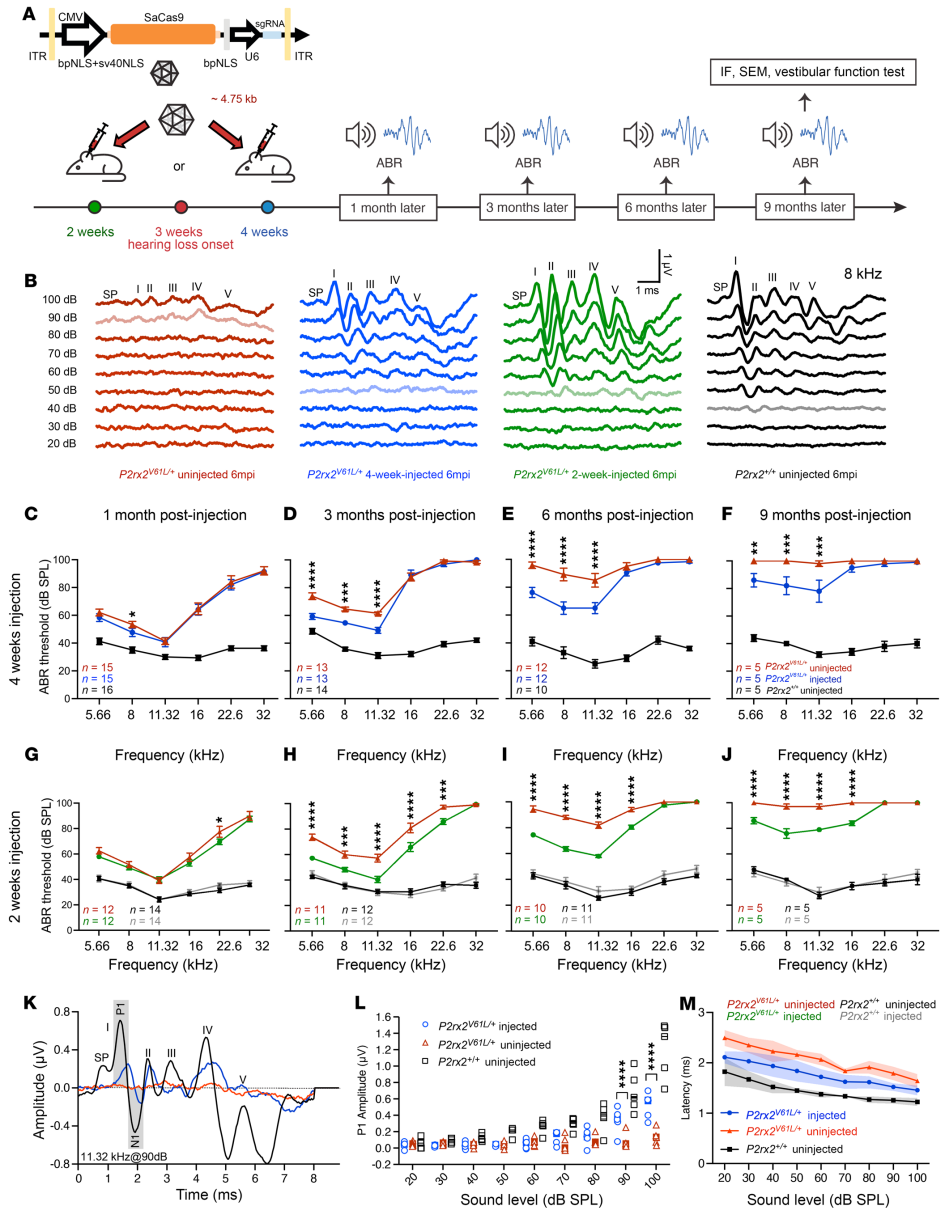
AAV2–SaCas9–sgRNA-1 adult and juvenile injections preserve auditory function in a *P2rx2^V61L/+^* mouse model of DFNA41. (**A**) Schematic diagram showing SaCas9 and sgRNA1 packaged into an AAV2 capsid, and illustrating the surgical intervention strategy. Hearing was tested at 1, 3, 6, and 9 months after injection (mpi). (**B**) Representative ABR waveforms were recorded at 6 months from an uninjected *P2rx2^V61L/+^* mouse (red), a 4-week–injected *P2rx2^V61L/+^* mouse (blue), a 2-week–injected *P2rx2^V61L/+^* mouse (green), and an uninjected *P2rx2^+/+^* mouse (black). Lighter traces indicate the threshold. (**C**–**F**) ABR thresholds of *P2rx2^V61L/+^* mice injected at 4 weeks old (blue), and uninjected *P2rx2^V61L/+^* littermates (red). ABR thresholds for uninjected *P2rx2^+/+^* mice littermates (black) tested at the same time points at 2, 4, 7 and 10 months of age. (**G**–**J**) ABR thresholds of *P2rx2^V61L/+^* mice injected at 2 weeks of age (green) and uninjected *P2rx2^V61L/+^* littermates (red), and injected and uninjected WT (*P2rx2^+/+^*) littermates (grey and black) tested at 1, 3, 6, and 9-months after injection. (**K**) Mean ABR waveforms in uninjected (red), injected (blue) *P2rx2^V61L/+^* 9 months after 4 week injection, and uninjected (black) *P2rx2^+/+^* at 90 dB 11.32 kHz. (**L**) Quantification of P1 amplitude at 11.32 kHz from 20 dB to 100 dB SPL for injected *P2rx2^V61L/+^* and uninjected *P2rx2^V61L/+^* mice at 9 months after injection at 4 weeks old compared with uninjected *P2rx2^+/+^* mice (black), with significant differences at 90 dB and 100 dB between injected (blue) and uninjected (red) *P2rx2^V61L/+^* groups. (**M**) Quantification of P1 latency at 11.32 kHz from 20 dB to 100 dB SPL for injected *P2rx2^V61L/+^*, uninjected *P2rx2^V61L/+^*, and uninjected *P2rx2^+/+^* mice at 9 months after injection at 4 weeks old. All data are presented as mean ± SEM. Two-way ANOVA determined significance with Bonferroni correction for multiple comparisons. *P < 0.05, **P < 0.01, ***P < 0.001, ****P < 0.0001.

To assess the therapeutic potential of AAV2–SaCas9–sgRNA-1, we aimed to mimic the clinical treatment scenarios by treating *P2rx2^V61L/+^* mice at 2 time points: (a) 4 weeks old (young adult) and (b) 2 weeks old (earliest detectable hearing). We injected 4 × 10^9^ vg AAV2–SaCas9–sgRNA-1 per ear via RWM + CF (Figure 5A). Auditory functions were evaluated at 1, 3, 6, and 9 months after injection using ABR and DPOAE across 6 frequencies from 5.66 to 32 kHz (Figure 5A).

In the 4-week-injection group, 1 month after injection, a significant 6 dB reduction in ABR threshold was detected at 8.00 kHz in injected ears compared with uninjected *P2rx2^V61L/+^* ears (Figure 5C). No significant difference in DPOAE thresholds was detected between injected and uninjected ears (Supplemental Figure 4A).

At 3 months after injection, treated *P2rx2^V61L/+^* ears showed substantial hearing preservation, whereas untreated ears had further deterioration across all frequencies. Treated ears had significantly reduced ABR thresholds at 5.66, 8.00, and 11.32 kHz, with an average reduction of 13 dB (Figure 5D). DPOAE threshold was reduced by 11 dB at 11.32 kHz (Supplemental Figure 4B).

Six months after injection, more significant hearing preservation was observed at 5.66, 8.00, and 11.32 kHz (Figure 5E). At this point, ABR thresholds in the untreated *P2rx2^V61L/+^* ears exceeded 85 dB across all frequencies, whereas injected ears had an average reduction of 21 dB at these frequencies (Figure 5E). DPOAE threshold at 11.32 kHz was reduced by 10 dB (Supplemental Figure 4C).

At 9 months after injection, hearing preservation persisted in treated ears (Figure 5F). Average ABR thresholds in untreated ears exceeded 95 dB across all frequencies, whereas injected ears had significant preservation at 5.66, 8.00, and 11.32 kHz, with an average ABR threshold reduction of 17 dB (Figure 5F). DPOAE thresholds at 11.32 kHz were reduced by 7 dB (Supplemental Figure 4D).

These findings demonstrate that AAV2–SaCas9–sgRNA-1 editing therapy provides robust and long-term preservation of hearing at specific frequencies in *P2rx2^V61L/+^* mice.

### Early genome editing therapy enhances the preservation of auditory function in P2rx2^V61L/+^ mice.

To determine if earlier intervention could result in more efficacious hearing rescue, we injected AAV2–SaCas9–sgRNA-1 in *P2rx2^V61L/+^* mice at P14 and subsequently evaluated auditory function. One month after injection, injected ears had a significant 8 dB reduction in ABR threshold at 22.6 kHz compared with uninjected *P2rx2^V61L/+^* ears (Figure 5G). No significant difference in DPOAE thresholds was observed between injected and uninjected ears (Supplemental Figure 5A).

At 3 months after injection, ABR thresholds in injected ears were significantly reduced at all frequencies except for 32 kHz, with reductions ranging from 16 dB at 5.66 kHz to 11 dB at 22.6 kHz, averaging 14 dB across the 5 frequencies (Figure 5H). DPOAE thresholds in injected ears were significantly reduced at 11.32 and 16 kHz by 17 and 14 dB, respectively (Supplemental Figure 5B).

Six months after injection, ABR thresholds had further deteriorated in uninjected ears, and injected ears had significant reductions at 5.66, 8.00, 11.32, and 16.00 kHz, with an average reduction of 21 dB (Figure 5I). DPOAE thresholds were significantly reduced by 16 dB at 11.32 kHz and by 6 dB at 16.00 kHz (Supplemental Figure 5C).

At 9 months after injection, uninjected ears exhibited profound hearing loss with ABR thresholds exceeding 95 dB across all frequencies. In contrast, injected ears had significant ABR threshold reductions at 5.66, 8.00, 11.32, and 16.00 kHz, averaging 17 dB (Figure 5J). DPOAE threshold was significantly reduced at 11.32 kHz by 7 dB in the injected ears (Supplemental Figure 5D).

Finally, at 12 months after injection, uninjected ears had ABR thresholds over 100 dB at all frequencies. Injected ears, however, had significant ABR threshold reduction at 8.00 and 11.32 kHz, averaging 16 dB (Supplemental Figure 6A). DPOAE threshold did not differ between injected and uninjected ears (Supplemental Figure 6B).

To evaluate how AAV2–SaCas9–sgRNA-1 injection affects normal hearing, we injected AAV2–SaCas9–sgRNA-1 into 2-week-old WT CBA mice and studied their hearing. The ABR and DPOAE thresholds in the injected WT mice were indistinguishable from WT mice without injection up to 12 months after injection (Figure 5, G–J, Supplemental Figure 5, A–D, and Supplemental Figure 6, A and B), demonstrating that the editing complex does not affect normal hearing, consistent with mutation-specific editing.

Consistent with ABR and DPOAE threshold improvement, significantly greater wave I P1 amplitudes at 11.32 kHz at 90 and 100 dB sound pressure levels (SPLs) were observed in 4-week-old injected *P2rx2^V61L/+^* ears 9 months after injection compared with untreated ears, in which P1 amplitudes were negligible (Figure 5, K and L, and Supplemental Figure 7). Additionally, in treated ears 9 months after injection at 4 weeks of age, wave I latency at 11.32 kHz across 20 to 100 dB SPL was reduced to a pattern more closely resembling that of WT ears compared with untreated ears (Figure 5M).

Based on the results, we conclude that AAV2–SaCas9–sgRNA-1 injection into the adult *P2rx2^V61L/+^* inner ear effectively preserves auditory function over the long term. Earlier intervention further enhances therapeutic efficacy by broadening the range of frequencies preserved.

### AAV2–SaCas9–sgRNA-1 editing therapy attenuates heightened sensitivity to NIHL in P2rx2^V61L/+^ mice.

**Figure 6:**
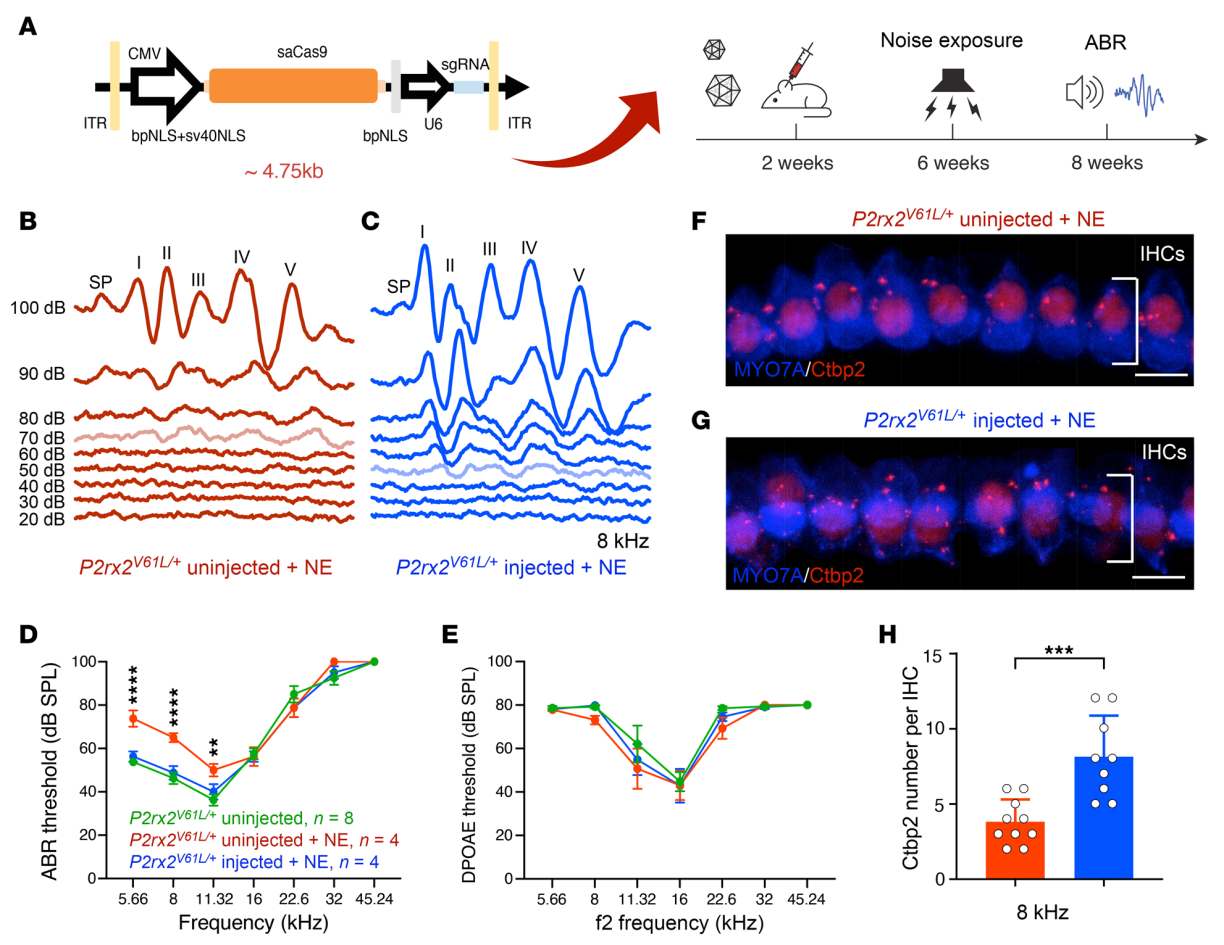
AAV2–SaCas9–sgRNA-1 injection attenuates increased sensitivity to NIHL in a *P2rx2^V61L/+^* mouse model of DFNA41. (**A**) Schematic representation of the experimental design. (**B** and **C**) Representative ABR waveforms at 8 kHz of the contralateral uninjected ear (B) and the injected ear (**C**) of a *P2rx2^V61L/+^* mouse after noise exposure. The ABR thresholds of 70 dB and 50 dB were detected in the uninjected (light red) and injected ears (light blue), respectively. (**D** and **E**) ABR thresholds (**D**) and DPOAE threshold (**E**) among different groups: injected and noise-exposed *P2rx2^V61L/+^* (blue), uninjected and noise-exposed *P2rx2^V61L/+^* (red), and uninjected *P2rx2^V61L/+^* without noise exposure (green). (**F** and **G**) Immunostaining of pre–ribbon synapse marker of CtBP2 of uninjected (**F**) and injected (**G**) *P2rx2^V61L/+^* mice followed by noise exposure (NE). MYO7A labels HCs. (**H**) Quantification and comparison of CtBP2 number showed a significant increase in the number of CtBP2^+^ synapses in the injected compared with uninjected ears after noise exposure. All data are presented as mean ± SEM. Significance was determined by 2-way ANOVA with Bonferroni correction for multiple comparisons for ABR and DPOAE and by unpaired Student’s *t* test for CtBP2. **P < 0.01, ***P < 0.001, ****P < 0.0001. Scale bars: 10 μm.

P2X2 receptors protect against NIHL by mitigating cation burden from high-intensity noise stimulation (56, 57). *P2rx2*-mutant mice are more susceptible to noise exposure (>95 dB), resulting in permanent threshold shift due to defects such as disruption of HC ribbon synapses (58). Indeed, *P2rx2^V61L/+^* mice exhibited greater sensitivity to NIHL than *P2rx2^+/+^* mice under identical noise exposure conditions (97 dB at the octave band of 1–20 kHz for 2 hours) (Figure 6, B–E, and Supplemental Figure 8, A–D), demonstrating remarkable similarities between mice and humans in terms of hearing deficits.

Because AAV2–SaCas9–sgRNA-1–mediated editing disrupts the V61L mutation responsible for increased NIHL sensitivity in *P2rx2^V61L/+^* mice, we hypothesized that this editing would protect the treated ears against NIHL. To test the hypothesis, we injected AAV2–SaCas9–sgRNA-1 into *P2rx2^V61L/+^* mice at P14. Four weeks later, the mice were exposed to 97 dB SPL at the octave band of 1–20 kHz for 2 hours, followed by a hearing test 2 weeks after noise exposure (Figure 6A). Controls included contralateral uninjected ears exposed to noise and *P2rx2^V61L/+^* mice without noise exposure.

In untreated *P2rx2^V61L/+^* ears, noise exposure led to significant hearing loss, as evidenced by elevated ABR thresholds at 5.66, 8.00, and 11.32 kHz compared with age-matched *P2rx2^V61L/+^* mice without noise exposure (Figure 6, B and D). In contrast, AAV2–SaCas9–sgRNA-1 injected ears had complete attenuation of NIHL, with the ABR thresholds virtually identical to those of nonexposed *P2rx2^V61L/+^* mice (Figure 6, C and D). No significant change in DPOAE thresholds was observed before or after noise exposure (Figure 6E), indicating that the noise did not affect the OHC function.

To further assess the optimal timing for genome editing intervention against noise sensitivity, we injected AAV2–SaCas9–sgRNA-1 into 4-week-old *P2rx2^V61L/+^* mice, followed by noise exposure 4 weeks later at 97 dB SPL at the octave band of 1–20 kHz for 2 hours, followed by hearing tests 2 weeks later (Supplemental Figure 8A). Again, injection in 4-week-old *P2rx2^V61L/+^* mice significantly attenuated ABR threshold shifts at 8.00 and 11.32 kHz compared with uninjected ears (Supplemental Figure 9, A and B). No significant changes in DPOAE thresholds or hair cell loss were observed after noise exposure (Supplemental Figure 9, C–E). Thus, editing in older *P2rx2^V61L/+^* mice partially protects against NIHL.

Ribbon synapses, essential for converting mechanical signals from HCs to electrical signals for auditory neurons, are highly vulnerable to noise exposure. CtBP2, a presynaptic protein, serves as a marker of synaptic integrity (59, 60). In noise-exposed WT mice, loss of ribbon synapses was observed despite no threshold shift, compared with nonexposed WT controls (Supplemental Figure 8, E–G). In contrast, injected *P2rx2^V61L/+^* ears exposed to noise exhibited significantly higher CtBP2 counts per IHC in the apex turn compared with uninjected noise-exposed ears (Figure 6, F–H). This finding demonstrates that editing in *P2rx2^V61L/+^* protected against synapse loss.

These results demonstrate that AAV2–SaCas9–sgRNA-1 treatment effectively attenuates heightened sensitivity to NIHL in *P2rx2^V61L/+^* mice, with improved outcomes after early intervention. This strongly supports a promising therapeutic strategy to mitigate NIHL in patients with *P2RX2* mutations.

### AAV2–SaCas9–sgRNA-1 treatment restores vestibular function in P2rx2^V61L/+^ mice.

Our previous study demonstrated that *P2rx2^V61L/+^* mice exhibited vestibular dysfunction (41). To assess whether AAV2–SaCas9–sgRNA-1–mediated CRISPR/Cas9 editing improves vestibular HC survival and subsequent vestibular functional recovery, we first evaluated AAV2 transduction efficiency in vestibular organs. We injected an AAV2-GFP vector into the cochleae of adult WT mice via the RWM+CF approach and examined GFP expression in vestibular HCs. Moderate GFP transduction was observed in vestibular HCs (Supplemental Figure 10), suggesting that AAV2–SaCas9–sgRNA-1 inner ear delivery could potentially preserve some vestibular function in *P2rx2^V61L/+^* mice.

**Figure 7:**
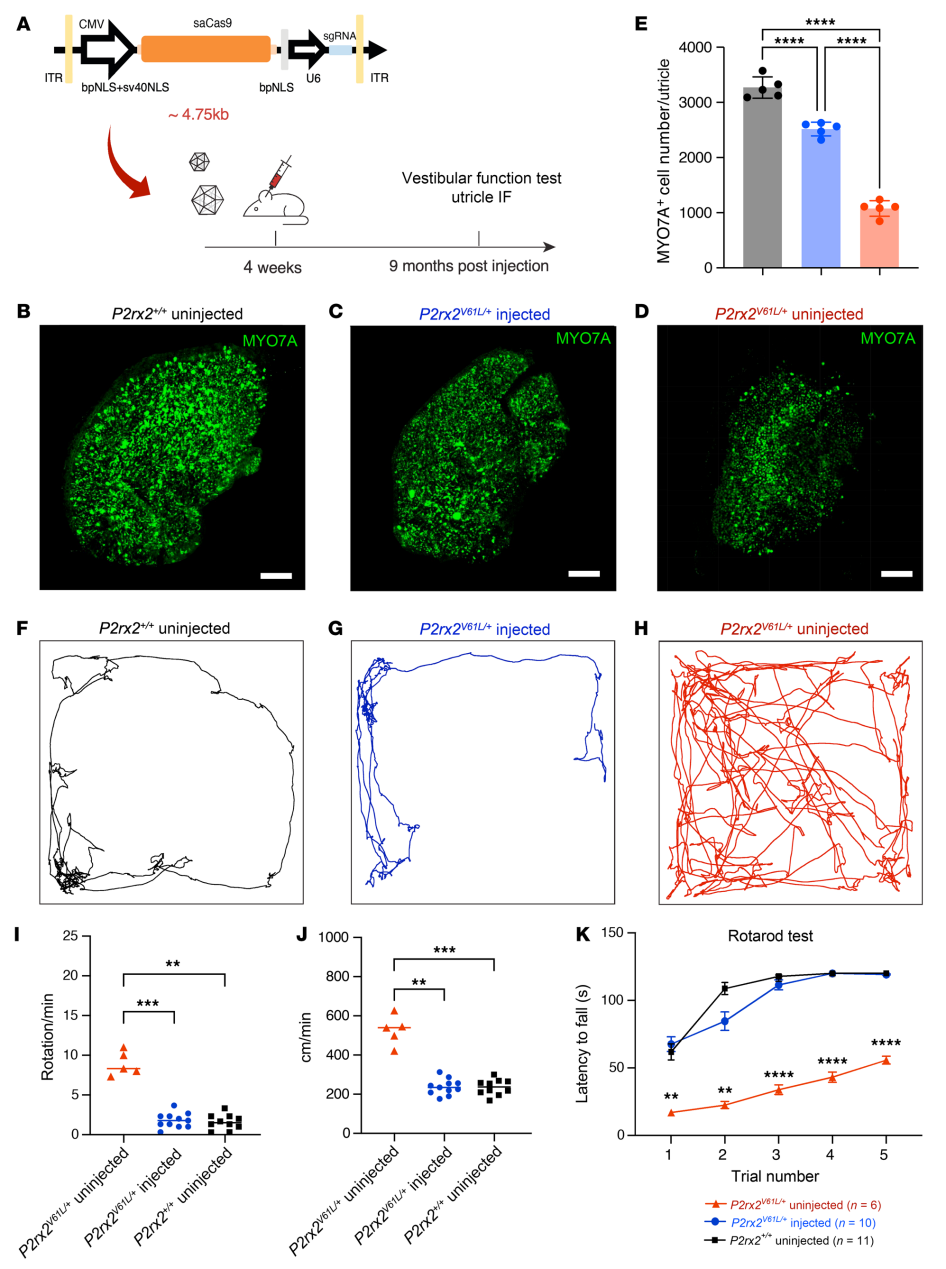
AAV2–SaCas9–sgRNA-1 adult injection rescues utricle HCs and vestibular function in the *P2rx2^V61L/+^* mouse model of DFNA41. (**A**) Schematic representation of the experimental design. (**B**–**D**) MYO7A-immunolabeled utricle of WT uninjected, *P2rx2^V61L/+^* injected, and *P2rx2^V61L/+^* uninjected ears at 9 months after injection at 4 weeks old. (**E**) MYO7A^+^ cell counts from WT utricles (black), *P2rx2^V61L/+^* injected (blue), and *P2rx2^V61L/+^* uninjected (red). *n* = 5. (**F**–**H**) Representative recording of open field test tracking the movements for 3 minutes for WT (**F**), injected (**G**), and uninjected (**H**) *P2rx2^V61L/+^* mice, 9 months after injection at 4 weeks old. (**I**) The number of full-body rotations per minute in the open field test among *P2rx2^V61L/+^* uninjected, *P2rx2^V61L/+^* injected, and WT groups. (**J**) Distance covered per minute for mice tested in the open field tests among *P2rx2^V61L/+^* uninjected, injected, and WT groups. (**K**) The result of rotarod performance in 5 trials at 9 months after injection of WT, uninjected, and injected *P2rx2^V61L/+^* mice. All data are presented as mean ± SEM. A 2-way ANOVA with Tukey’s multiple comparison test. **P < 0.01, ***P < 0.001, ****P < 0.0001. Scale bar: 50 μm. IF, immunofluorescence. NLS, nuclear localization signals.

To further investigate feasibility, we evaluated the impact of AAV2–SaCas9–sgRNA-1–mediated CRISPR/Cas9 editing on utricle HC survival. We quantified utricle HC numbers at 9 months after injection in 4-week-old *P2rx2^V61L/+^* mice (Figure 7, A–E). In uninjected *P2rx2^V61L/+^* ears, approximately 70% of utricle HCs were lost compared with the WT utricle, as shown by immunolabeling and quantification (Figure 7, B, D, and E). In contrast, in injected *P2rx2^V61L/+^* ears, significantly more HCs were preserved, with approximately 30% more HCs compared with the uninjected utricle (Figure 7, C and E).

To assess vestibular function, we performed open field and rotarod tests 9 months after injection. In the open field test, untreated *P2rx2^V61L/+^* mice displayed hyperactive behavior, including increased activity across the entire field and more frequent full-body rotations compared with untreated WT mice (Figure 7, F and H). In contrast, injected *P2rx2^V61L/+^* mice showed normalized behavior, preferring to explore along the border of the chamber with minimal full-body rotations, similar to WT mice (Figure 7G). Treated *P2rx2^V61L/+^* mice also had decreased velocity and travel distance compared with untreated *P2rx2^V61L/+^* mice, aligning more closely with WT levels (Figure 7, I and J).

In the rotarod test, uninjected *P2rx2^V61L/+^* mice displayed poor rotarod performance in maintaining their balance within the allocated time (Figure 7K). In contrast, injected *P2rx2^V61L/+^* mice showed partially improved rotarod performance, comparable to that of WT mice (Figure 7K).

Collectively, these results from the open field and rotarod tests indicate AAV2–SaCas9–sgRNA-1 treatment effectively rescues vestibular function in *P2rx2^V61L/+^* mice over the long term.

### In vitro screening of genome editing strategies targeting the human P2RX2 V60L mutation in patient-derived hiPSCs.

**Figure 8:**
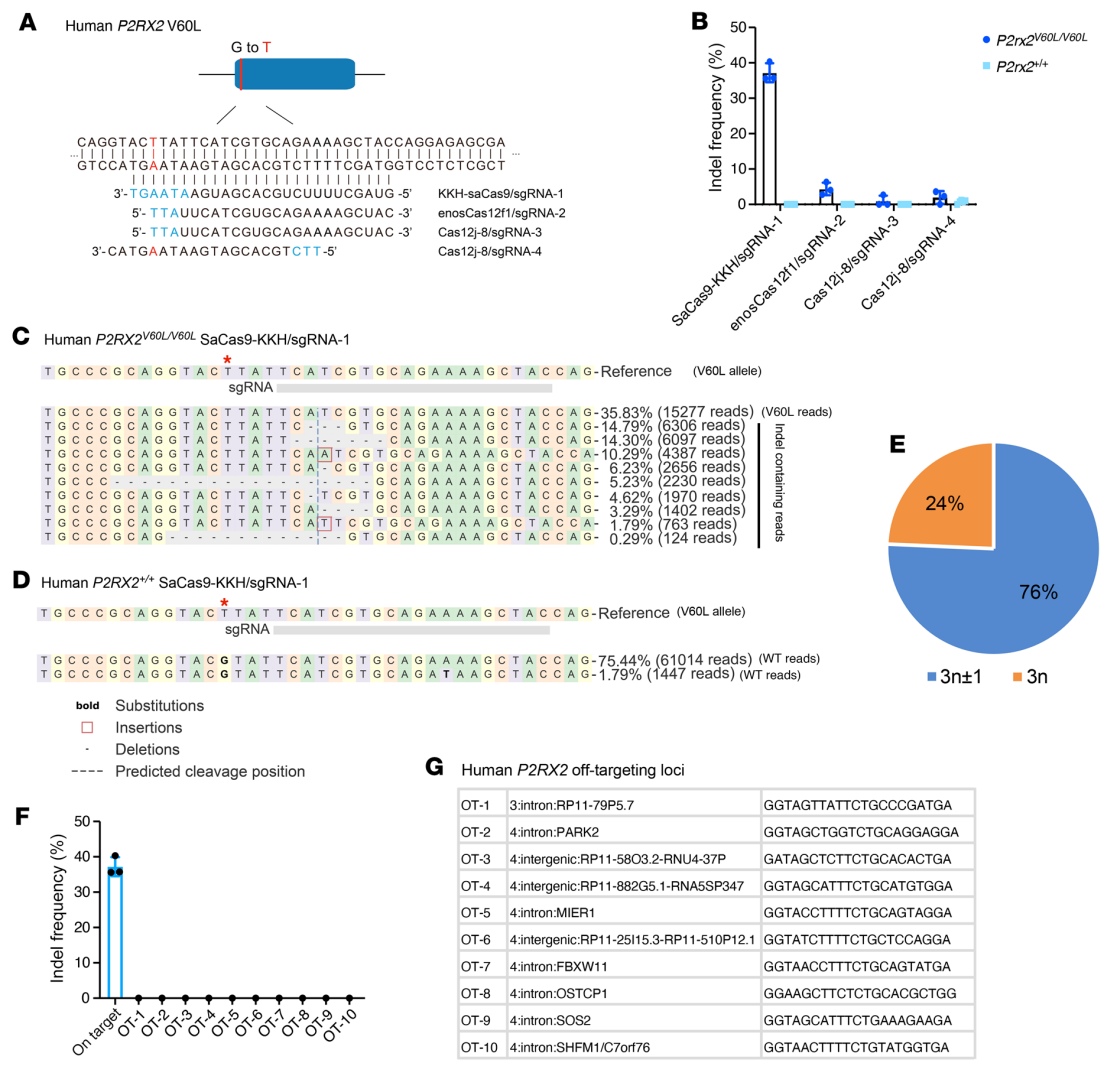
*P2RX2^V60L^* allele-specific genome editing in patient-derived hiPSCs using compact CRISPR systems. (**A**) Sequence of the human *P2RX2* V60L mutation locus and the sgRNAs designs. The mutation is displayed in red. Protospacer-adjacent motifs are displayed in blue. (**B**) Bar chart of the indel frequency after genome editing using different CRISPR systems on hiPSCs with homozygous mutations (*P2RX2^V60L/V60L^*) and WT hiPSCs (*P2RX2^+/+^*). *n* = 3. Each dot represents a unique sequencing reaction. Values and error bars reflect mean ± SD. (**C** and **D**) Representative NGS results from KKH–SaCas9–sgRNA-1–edited *P2RX2^V60L/V60L^* and *P2RX2^+/+^* hiPSCs. No indels were detected in the normal *P2RX2* allele. Red asterisks in the reference sequence indicate the V60L mutation nucleotide. (**E**) Pie chart showing the out-of-frame (3n ± 1) ratio in the indel profile. (**F**) Quantification of indel frequency of potential off-target sites from AAV2–KKH–SaCas9–sgRNA-1–edited human cells. (**G**) The sequences of potential off-target genetic loci of KKH–SaCas9–sgRNA-1 in the human genome. None of these loci was associated with hearing function.

Our ultimate goal is to develop an editing strategy to rescue hearing in patients with DFNA41. To design gRNAs to abolish the *P2RX2* V60L mutation in the human genome, we tested gRNA with different editors in patient-derived human induced pluripotent stem cells (hiPSCs) harboring the *P2RX2 c.178G > T* mutation (55). We screened various compact CRISPR systems that can be accommodated in a single AAV vector, including SaCas9-KKH (61), enosCas12f1 (62), and Cas12j-8 (63) (Figure 8A). Plasmids encoding CRISPR nucleases and sgRNAs were introduced into hiPSCs using nucleofection. NGS analysis revealed robust indel generation in cells edited with SaCas9-KKH/sgRNA-1, with an indel frequency of 37.9% ± 2.3% (Figure 8B). Indel profiling further revealed that in reads edited with SaCas9-KKH/sgRNA-1, a majority of indels were frameshift mutations (Figure 8, C and E).

We performed an editing study in hiPSCs derived from a healthy control individual. We did not detect indels in the control hiPSCs (Figure 8, B and D), demonstrating that our gRNA design is mutation specific and does not edit the normal allele. In comparison, the other compact CRISPR systems exhibited substantially lower on-target genome editing efficiency, with enosCas12f1/sgRNA-2 at 4.6% ± 2.2% and Cas12j-8 at less than 3% (Figure 8B). Neither enosCas12f1/sgRNA-2 nor Cas12j-8 edited the WT allele (Figure 8B). We further tested the top 10 potential off-target loci in the human genome, and NGS revealed no indels (Figure 8, F and G). Collectively, these data demonstrate that we have identified a gRNA for SaCas9-KKH that enables efficient and specific editing of human *P2RX2* c.178G > T (V60L) dominant mutation, with minimal off-target effects.

## Discussion

Since the first gene therapy study in a mouse model of genetic hearing loss (7), numerous gene and genome editing therapies have shown success in various mouse models (3, 13, 21–23, 25, 26, 30–32). Despite promising results, most studies—except for those of *Otof* (38), *Tmprss3* (13), and *Mir96* (39)—were conducted in neonatal mice with immature inner ears. In mice, hearing begins at P14, cochlear maturation completes by P20 (34), and HC–neuron synapses mature around P28 (35). In humans, inner ear is fully mature before birth (36, 37). Thus, translating these approaches to humans requires effective intervention in a fully mature inner ear.

DFNA41 is caused by mutations in *P2RX2*, which encodes the P2X2 receptor, an ATP-gated trimeric cation channel composed of an extracellular ATP-binding domain, two α-helical transmembrane segments (TM1 and TM2), and intracellular N- and C-termini (64). ATP binding activates the receptor and opens the ion channel (65). Four mutations have been linked to DFNA41: c.601G>A (p.D201Y) in a Japanese family (66); c.1057G>C (p.G353R) in an Italian family (67); c.1048T>G (p.X350E) in an Iranian family (68); and c.178G>T (p.V60L) in 2 unrelated Chinese families (55, 69). Despite affecting different receptor domains, all mutations result in delayed-onset, progressive autosomal dominant hearing loss (67, 70, 71).

We produced a knock-in *P2rx2^V61L/+^* mouse model carrying a c.179G>C (V61L) mutation, which mimics the human *P2RX2* V60L variant and causes a dominant gain-of-function effect (40). This model reproduces the delayed-onset, progressive hearing loss and increased NIHL susceptibility seen in patients with DFNA41. P2RX2 possesses ATPase activity, and the V60L mutation disrupts its activation (42), making the model ideal to evaluate a genome editing strategy to abolish the dominant mutation and preserve hearing. AAV2 was used because it efficiently transduces most adult HCs. Similar phenotypes in patients with DFNA41 support a general applicability of our intervention.

We screened gRNAs for different editors and identified an SaCas9 gRNA with efficient on-target editing and minimal off-target effects. In vivo AAV2–SaCas9–sgRNA-1 injection in 4-week-old *P2rx2^V61L/+^* mice achieved >25% editing efficiency in HCs, as confirmed by NGS of genomic DNA and transcripts. Off-target effects in vivo were negligible. Our previous studies demonstrated that efficient editing in neonatal HCs rescues hearing in multiple mouse models of genetic hearing loss (20, 22, 72), suggesting the *P2rx2* gRNA could preserve hearing in adult mice, provided the HCs remain intact.

We obtained robust safety data from local AAV2–SaCas9–sgRNA-1 injection in adult mice. AAV-mediated delivery can lead to unwanted genomic integration (48, 52). Although AAV2–SaCas9–sgRNA-1 was integrated into genomic DNA in cultured cells, the integration rate is dose dependent. At the in vivo dose used, no AAV genomic integration was detected, markedly reducing the risk associated with inner ear editing. Additionally, Cas9 expression driven by CMV promoter is transient, with undetectable Cas9 expression after 12 weeks. If a similar expression pattern occurs in humans, it could substantially reduce a key safety concern for genome editing therapies.

Dominant hearing loss from gain-of-function or dominant-negative mutations can be addressed by genome editing to selectively disrupt the mutant allele. These conditions typically present as delayed-onset, progressive hearing loss, offering a treatment window across different age groups. Timing of intervention is likely to affect efficacy and durability of therapeutic benefit.

In 4-week-old *P2rx2^V61L/+^* mice with mature inner ears, hearing preservation became evident at 3 frequencies by 3 months after injection compared with contralateral ears. From 6 to 9 months after injection, thresholds in uninjected ears deteriorated, eventually exceeding detection limits by 9 months, indicating profound hearing loss. In contrast, injected ears had a slower rate of thresholds elevation, with average ABR improvements of 13 dB, 21 dB, and 17 dB across 3 frequencies and DPOAE threshold reductions of 11 dB, 10 dB, and 7 dB at 11.32 kHz.

Previous studies have shown that AAV-mediated expression begins about 1 week after injection (73, 74). In *P2rx2^V61L/+^* mice injected at 4 weeks, SaCas9 expression peaked at 4 weeks later (Figure 2F), likely after high-frequency hearing loss had developed, thus limiting rescue potential. To test this, we injected 2-week-old *P2rx2^V61L/+^* mice and found improved efficacy: the rescue of ABR thresholds was extended to 5.66, 8.00, 11.32, and 16.00 kHz, with DPOAE thresholds extended to 11.32 and 16 kHz. The effect persisted for 12 months. Although we used ABR and DPOAE to evaluate auditory functions, they have limitations. Future studies incorporating additional tests, such as the startle reflex and prepulse inhibition, should measure auditory recovery more comprehensively.

The enhanced efficacy of early injection may result from a greater number of HCs available for editing and/or greater susceptibility to editing in younger cells. The expanded rescue to 16 kHz with 2-week injection strongly suggests more HCs in the frequency region are amenable to editing and functional recovery. If editing efficiency was enhanced by 2-week injection, we would expect more efficacious hearing rescue at 5.66, 8.00, and 11.32 kHz, yet outcomes at these frequencies were comparable to 4-week injections, indicating that early injection does not improve editing efficiency. Thus, better high-frequency recovery likely reflects greater HC availability for editing at high frequency. This comparative study supports the sustained therapeutic benefit of genome editing in both juvenile and adult inner ears, with early intervention offering additional benefits. In patients with DFNA41, hearing loss typically begins in the second decade and worsens progressively over decades (55). Our findings support early, even presymptomatic, intervention to maximize efficacy and highlight the need to further improve editing efficiency for better outcomes.

Despite notable hearing preservation after injection in 4-week-old *P2rx2^V61L/+^* mice, we did not observe recovery in high-frequency hearing. This may reflect mechanical damage from RWM+CF delivery, which can affect the vulnerable basal region (75–78). Additionally, base-turn HCs may have degenerated at the time of editing, because high-frequency hearing loss in *P2rx2^V61L/+^* mice is detectable by P21 (41), before the time of 4-week injection. The lower titer of AAV2–SaCas9–sgRNA-1 (4.46 × 10¹² vg/mL) compared with AAV2-GFP (1 × 10¹³ vg/mL) may have also reduced transduction efficiency in the basal turn (Supplemental Figure 3).

Patients with DFNA41 have increased vulnerability to NIHL (55). In the inner ear, sound transduction is driven by the endocochlear potential (approximately +100 mV) and the negative membrane potential of the HCs (79). Elevated sound levels trigger ATP release into the cochlea, activating P2X2 receptors and dampening transduction and synaptic transmission (58). *P2rx2^V61L/+^* mice reproduce this phenotype, exhibiting heightened vulnerability to NIHL at >95 dB, resulting in permanent hearing loss primarily due to IHC ribbon synapse loss (55, 58). Genome editing at 2 weeks of age completely attenuated NIHL hypersensitivity: ABR thresholds normalized, and ribbon synapse loss was rescued. Editing at 4 weeks also remarkedly attenuated NIHL hypersensitivity (Supplemental Figure 9), though less completely. These results demonstrate editing treatment efficacy for DFNA41-associated phenotypes and highlight improved outcomes with early intervention.

Cation absorption via P2RX2 has also been identified in vestibular transitional cells of the semicircular canal ampulla, the crista ampullaris, utricular, and saccular macula (56, 80). P2RX2 and P2RX4 co-expression protects against high-intensity stimulation–induced utricule HC damage (81). In this study, genome editing rescued vestibular dysfunction in *P2rx2^V61L/+^* mice, demonstrating effective AAV-mediated targeting of adult vestibular HCs. These findings further support editing therapy for vestibular dysfunction associated with genetic hearing loss, such as in Usher syndrome.

Vestibular dysfunction has not been reported in patients with DFNA41, possibly because mild deficits go undetected without comprehensive testing. Humans may also possess stronger central compensatory mechanisms and rely on multiple sensory inputs, including vision, proprioception, and vestibular function, for balance, which may mask subtle dysfunction. Alternatively, patients with DFNA41 may not exhibit vestibular deficits seen in mutant mice, suggesting species-specific differences. We are currently conducting a natural history of patients with DFNA41, including vestibular function assessment.

At the cellular level, adult editing in *P2rx2^V61L/+^* mice promotes HC survival and preserves HC stereocilia integrity, likely underlying the observed hearing rescue. However, by 9 months after injection, HCs in the basal turn remained lost, possibly due to prior degeneration or inefficient editing. Future careful assessment of the status of HCs at the time of editing will inform us about the relationship among the status of HCs, editing, and hearing rescue. Additionally, as *P2RX2* is also expressed in non-HC types, editing in those cells may contribute to the therapeutic effect. Future studies using cell-type specific targeting and single-cell transcriptomic profiling will help delineate the cellular and molecular mechanisms driving functional rescue and to further refine therapeutic strategies.

For the human *P2RX2* c.178G>T mutation, we screened compact CRISPR systems—including SaCas9-KKH, enosCas12f1, and Cas12j-8—all suitable for a single AAV vector. However, NGS analysis revealed robust indel formation only with SaCas9-KKH/sgRNA-1, whereas enosCas12f1/sgRNA-2 and Cas12j-8 were ineffective, likely due to sgRNA performance differences in the human genome. Notably, the identified SaCas9-KKH gRNA supports the initiation of safety studies for clinical translation.

A key question is how effectively editing-mediated hearing rescue in the *P2rx2^V61L/+^* mouse can translate to humans. Recently, we conducted a successful clinical trial demonstrating robust hearing restoration using gene therapy of *OTOF* in patients with DFNB9, which closely mirrored mouse model outcomes (82, 83), underscoring strong translatability in phenotype, AAV delivery, safety, and efficacy. Given the reproducible *P2rx2^V61L/+^* mouse phenotypes of DFNA41 and successful AAV2-mediated editing in the mature inner ear, our study provides a compelling foundation for advancing toward clinical application. Based on the results, we have initiated an investigational new drug–enabling study to move our work into the clinic.

## Methods

### Sex as a biological variable.

Our study examined male and female animals. Similar findings are reported for both sexes.

### Mice.

*P2rx2^V61L/+^* mice were maintained on a CBA/J background. A female mouse carrying a *P2rx2* c179 G>C (p.V61L) was used for colony expansion by mating with a WT *P2rx2^+/+^* male mouse, as described previously (41). The mice were housed in groups of 2–4 per cage on a 12:12 hour–light-dark cycle and allowed free access to food and water. All the animals were maintained under standard conditions with a room temperature range of 22°C ± 2°C and humidity range 55% ± 10%.

### Plasmid construction.

U6-sgRNA sequence and SaCas9 (Addgene, 61591) (84) cDNAs were acquired from Addgene. Vectors for in vitro screening were constructed via Gibson assembly (New England Biolabs, E2611S) based on pMax-SpCas9 (85, 86). AAV vectors are based on pX601 (Addgene, 61591) (84). Plasmids encoding recombinant AAV genomes were cloned by Gibson assembly. All plasmids were purified using Plasmid Plus Miniprep or Maxiprep kits (Qiagen).

### AAV production.

AAV plasmids containing CMV-SaCas9-PA cassette, U6-sgRNA-1, or U6-sgCtrl cassette, were sequenced before packaging (Massachusetts General Hospital DNA Core) into AAV2/2 (Supplemental Figure 1). AAV vectors were produced by the Mass Eye and Ear vector core. The vector titer was 4.46 × 10^12^ vg/mL for AAV2–SaCas9–sgRNA-1, as determined by qPCR specific for the ITR of the virus.

### HC isolation and NGS analysis.

Cochleae were harvested with the sensory epithelia dissociated using needles under a microscope (Axiovert 200M, Carl Zeiss). Inner ear tissue was immersed in 1 μM FM 1-43FX (Thermo Fisher Scientific, F35355) for 15 seconds at room temperature in the dark, then washed by Dulbecco’s PBS. The sensory epithelia were treated with 100 μL of 0.05% trypsin-EDTA (Thermo Fisher Scientific, 25300054) for 20 minutes. During incubation, the tissue was carefully dispersed into small or single-cell clusters using a 200 μL Eppendorf pipette tip. Cells were transferred into a 10 mm culture dish and placed under a fluorescent microscope (Zeiss) equipped with a camera. FM 1-43FX–labeled HCs were collected by a 10 indel Eppendorf pipette tip, with approximately 300 cells collected from single cochlea. HCs were transferred into a PCR tube and lysed by 5 μL QuickExtract DNA Extraction Solution (Lucigen) for genomic DNA extraction. The cell lysis was used for genomic PCR amplification using NEBNext Ultra II Q5 Master Mix (New England Biolabs, M0544S) with the following program: 1 cycle: 98°C for 5 minutes; 42 cycles: 98°C for 15 seconds, 60°C 20 for seconds, 72°C for 10 seconds; and 1 cycle: 72°C for 4 minutes; 4°C. From 400 to 800 ng of purified PCR product was used for Next-Generation Sequencing (NGS). Samples were sequenced by the Massachusetts General Hospital Center for Computational and Integrative Biology DNA Core to visualize CRISPR variants.

### Genome editing in vitro.

LONZA 4D-Nucleofector was used for nucleofection of fibroblasts. Cells were digested by trypsin-EDTA (0.05%) (Thermo Fisher Scientific) and dispersed into single cells. A total of 100,000 cells were resuspended in 20 μL of P3 reagent of the P3 Primary Cell 4D-Nucleofector X Kit S (Lonza Bioscience, V4XP-3032) and nucleofected by program EH-100. Cells were then treated with puromycin 24 hours later for 2 days. Four days after nucleofection, genomic DNA was collected using QuickExtract DNA Extraction Solution (Lucigen) for PCR using NEBNext Ultra II Q5 Master Mix (New England Biolabs, M0544S), with purified PCR product used for NGS. Individual SEQ files were analyzed by CRISPResso2 (http://crispresso.pinellolab.org/submission) as instructed (87). The sgRNA protospacer sequences can be found in Supplemental Table 3.

### In vivo surgery.

The RWM+CF was performed in mice at P14 or P28. The mice were anesthetized using intraperitoneal injections of ketamine (100 mg/kg) and xylazine (10 mg/kg). The postauricular area hair was shaved from 150% of the surgical area and disinfected with 10% povidone-iodine and 70% ethanol for 3 cycles. The AAV-GFP (Vector Laboratories, 7004) or AAV2–SaCas9–sgRNA-1 was injected into the inner ears of *P2rx2^V61L/+^* or *P2rx2^+/+^* mice. A volume of 1–1.2 μL was injected into each cochlea.

### Confocal immunofluorescence.

Cochleae were extracted, perfused with 4% paraformaldehyde, postfixed for 12 hours at 4°C, and decalcified in 0.12 M EDTA for 48 hours. Samples were dissected into 2–3 pieces spanning apex to base and stained with the following antibodies: (a) mouse anti-CtBP2 (1:200; BD Biosciences, 612044) and (b) rabbit anti-MYO7A (1:100; Proteus Biosciences, 25-6790), followed by Alexa Fluor–conjugated secondary antibodies (1:1,000). Confocal Z-stacks were acquired using high-numerical-aperture objectives and ×2 digital zoom with 0.30 μm z-steps.

### ABR/DPOAE measurements.

ABRs and DPOAEs were recorded to test auditory ability. ABRs were elicited by tone pips at 5.66, 8.00, 11.32, 16.00, 22.60, and 32.00 kHz (5 ms duration; 0.5 ms cos^2^ rise–fall; alternating polarity; *n* = 512 sweeps each). ABR threshold was defined by visual inspection of stacked waveforms at 20–100 dB in 5–10 dB steps. Wave 1 amplitudes were extracted using a semiautomated algorithm. Subsets of cases were analyzed by a second investigator blinded to the experimental treatment. DPOAEs were evoked using f1 and f2 tones (f2 matching ABR frequencies; f2/f1 = 1.2; L1 = L2 + 10 dB, 5 dB steps). The 2f1–f2 response was extracted from ear canal sound pressure. Threshold was computed by interpolation as the f2 producing a DPOAE of 5 dB SPL.

### Noise exposure.

Awake mice were placed in a small wire mesh cage without restraints directly below the acoustic horn in a reverberant exposure chamber with a sound-delivery loudspeaker. A 1–20 kHz noise band was delivered at 97 dB SPL for 2 hours. The calibration was processed before each exposure session. The variation of sound level was within 1–1.5 dB.

### Vestibular test.

For the open-field test, awake mice were placed in a 38 cm × 38 cm arena positioned inside a sound chamber with overhead LED lighting, all inside a dimmed room. The mice were tested 1 at a time inside the square open field and allowed to explore for 3 minutes. Behavior was recorded by a Canon EOS R5 camera and tracking videos were analyzed by EthoVision XT, measuring the traveled distance and velocity. Raw measurements for the traveled distance were recorded in centimeters. We recorded videos We recorded videos during daytime hours from 8:00 a.m. to 5:00 p.m. to ensure mice adapted to their new environment. Open-field assessments were conducted by tester blinded to the experiments, and the same tester performed the experiments throughout the day.

The rotarod test was conducted to assess the mice’s performance on a rod that began rotating at 5 rpm for 5 minutes to familiarize mice with the equipment. The following day, the animals were placed on the rods for a total of 5 trials, with a 3 minute resting period between each trial. The same tester conducted all the experiments throughout the day. The duration of time the animals could remain on the rod before falling off was recorded for each trial.

### Statistics.

Statistical analysis was performed with Prism 10 software (GraphPad). Data are presented as mean ± SEM or SD. *P* values less than 0.05 were considered statistically significant. All group sizes, statistical tests, and *P* values are reported in the figures or the figure legends.

### Study approval.

All procedures were performed in accordance with NIH guidelines for use and care of laboratory animals and were approved by the Massachusetts Eye and Ear IACUC (protocol 2022N000065).

### Data availability.

The NGS data are available with the Sequence Read Archive accession number: SUB15099459. Values for all data points in graphs are reported in the Supporting Data Values file.

## Author contributions

ZYC and XZL conceptualized and supervised the study. WW, WZ, YS, XZL, and ZYC contributed to project administration and conducted the investigation. WW and WZ performed the experiments. SS and AMA conducted auditory function testing. WW and FSR conducted vestibular function testing. WW and APR performed scanning electron microscopy. WW, WZ, YQ, WD, and YK contributed to methodology development and data analysis. WW, WZ, YS, XZL, and ZYC wrote the manuscript. WW, WZ, SS, AMA, FSR, APR, KW, YQ, WD, YK, AAI, YS, XZL, and ZYC reviewed and edited the manuscript.

WW and WZ contributed equally to this work and are co-first authors. Authorship order between them was determined based on overall contribution to experimental design, data generation, and manuscript preparation.

## Supplementary Material

Supplemental data

Supporting data values

## Version 1 08/14/2025

In-Press Preview

## Version 2 10/15/2025

Electronic publication
