# Supplementary material for: Single-dose genome editing therapy rescues auditory and vestibular functions in adult mice with DFNA41 deafness
Source: J Clin Invest. 2025 Aug 14;135(20):e187872. doi: 10.1172/JCI187872 (PMC12520686; doi:10.1172/JCI187872)
Supplement: Supplemental data [file jci-135-187872-s292.pdf]

Supplementary Material for

Single-Dose Genome Editing Therapy Rescues Auditory and Vestibular Functions in Adult Mice with DFNA41 Deafness

Wei Wei *et al.*

Correspondence should be addressed to Z.Y.C (zheng-yi_chen@meei.harvard.edu), X.Z.L (x.liu1@med.miami.edu), and Y.S (yilai_shu@fudan.edu.cn)

The PDF file includes:

Supplemental Figure 1 to 10

DNA sequence of AAV-CMV-SaCas9-sgRNA-1.

Tables S1 to S3

Supplementary Methods

Figure S1

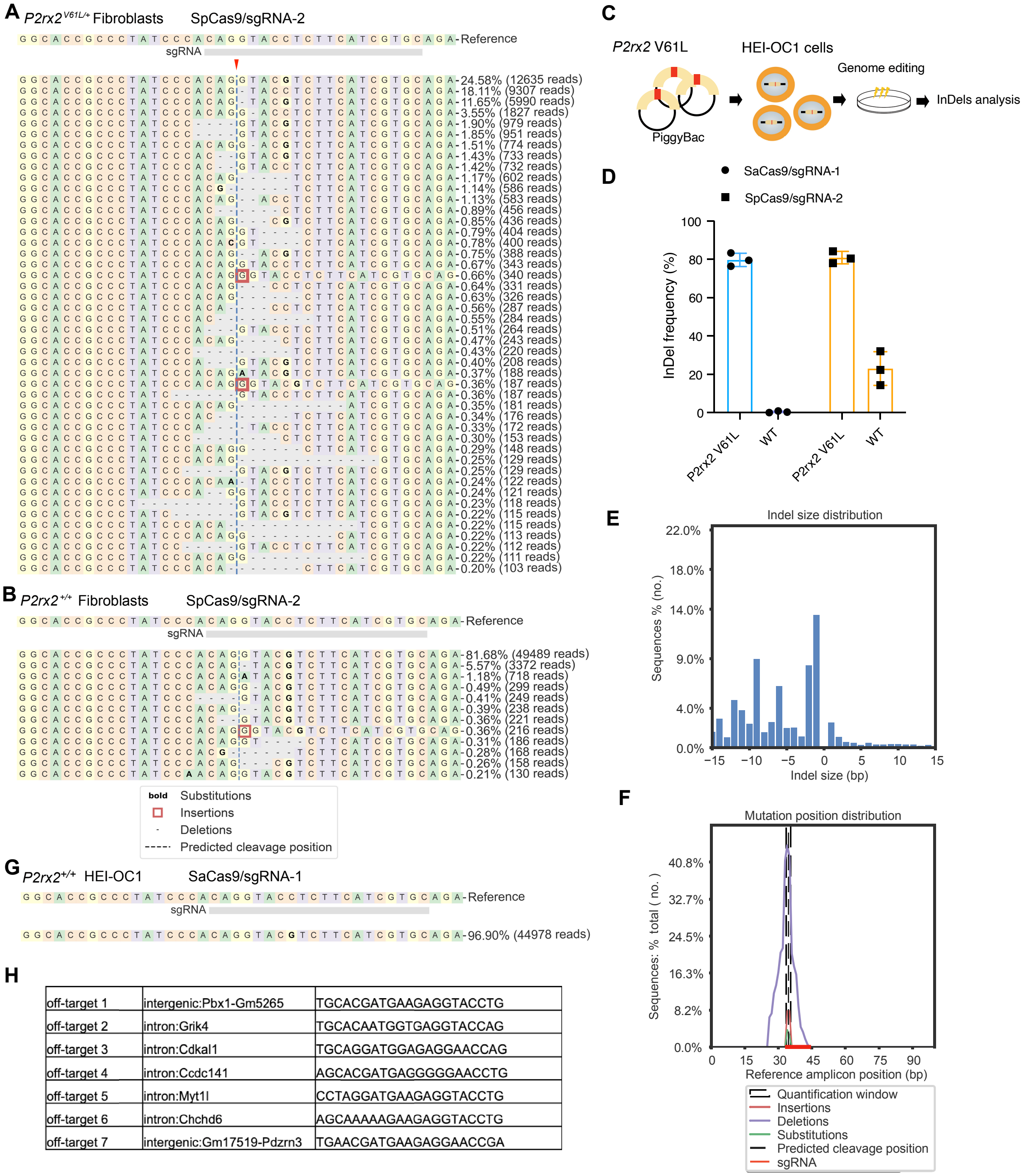

off-target 1	intergenic:Pbx1-Gm5265	TGCACGATGAAGAGGTACCTG
off-target 2	intron:Grik4	TGCACAATGGTGAGGTACCAG
off-target 3	intron:Cdkal1	TGCAGGATGGAGAGGAACCAG
off-target 4	intron:Ccdc141	AGCACGATGAGGGGGAACCTG
off-target 5	intron:Myt1l	CCTAGGATGAAGAGGTACCTG
off-target 6	intron:Chchd6	AGCAAAAAGAAGAGGTACCTG
off-target 7	intergenic:Gm17519-Pdzrn3	TGAACGATGAAGAGGAACCGA

Supplemental Figure 1. Targeting *P2rx2* V61L mutation with different CRISPR nuclease systems. (**A** to **B**) Representative NGS results from SpCas9/sgRNA-2 edited *P2rx2*$^{V61L/+}$ (A) and WT (B) primary fibroblasts. The red arrow indicates the double-

stranded DNA-cutting site. (**C**) Schematic overview of *P2rx2* V61L HEI-OC1 cell line establishment using the PiggyBac system and a genome editing procedure. (**D**) Quantification of the InDel frequency in *P2rx2* V61L and WT HEI-OC1 cells after genome editing using SpCas9/sgRNA-2 and SaCas9/sgRNA-1. Error bar represents SD. (**E** to **F**) InDel profiles from SpCas9/sgRNA-2 edited *P2rx2* V61L HEI-OC1 cells. Minus numbers represent deletions, plus numbers represent insertions. (**G**) Representative NGS results from SaCas9/sgRNA-1 edited WT HEI-OC1 cells. (**H**) The potential off-target genetic loci of SaCas9/sgRNA-1 in mouse genome. None of these loci were associated with hearing function.

Figure S2

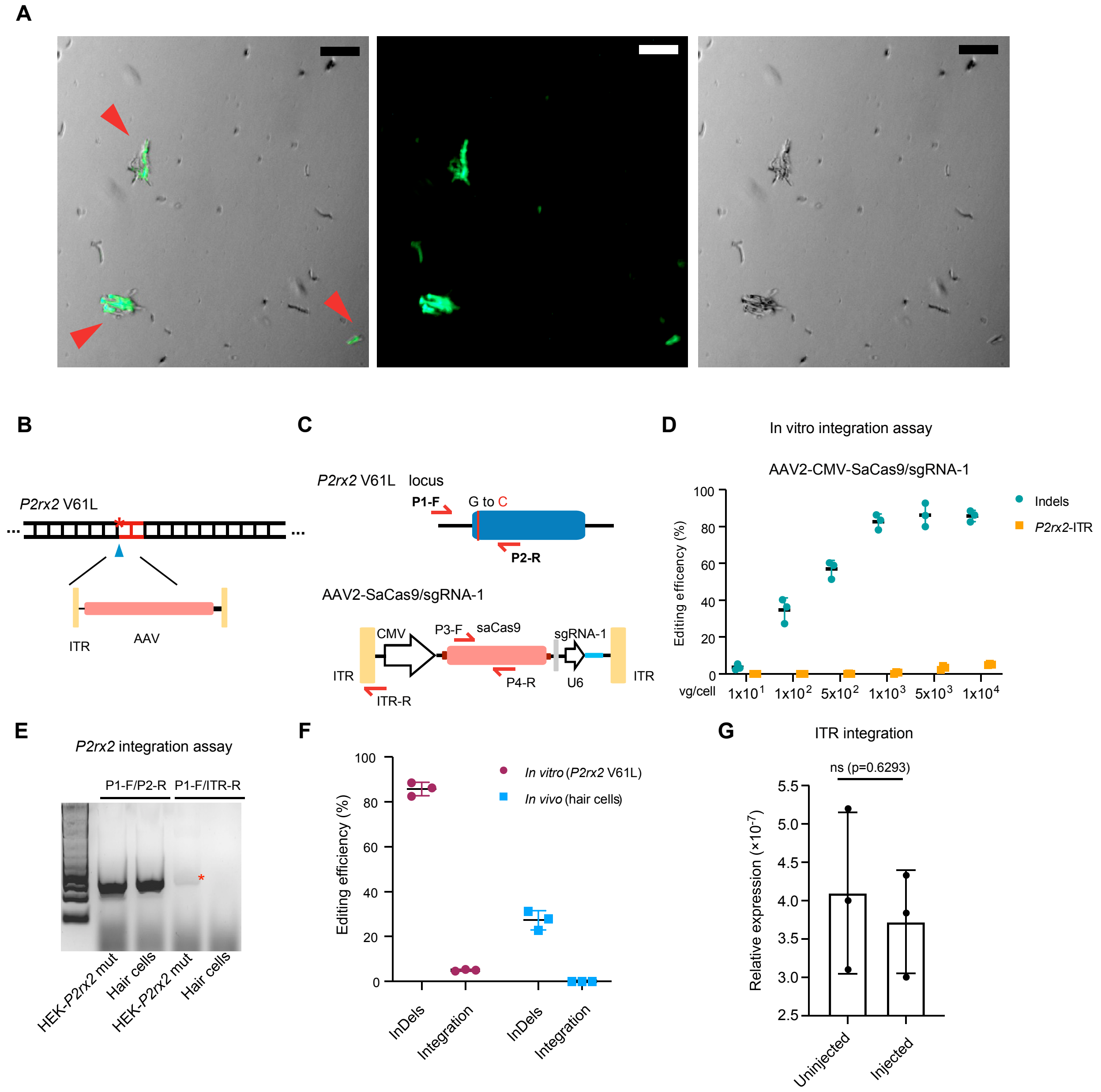

Supplemental Figure 2. Safety assessment of AAV-mediated genome editing in adult mice. (**A**) Representative images of FM1-43FX labeled hair cells after digestion of cochlear tissues. FM1-43FX labeled hair cells are shown in green (red arrowheads). (**B**) Schematic overview of the AAV vector integration at the double strands DNA break (DSB) site. The red asterisk indicates *P2rx2* V61L mutation site, blue arrawhead

indicates the DSB site. (**C**) Schematic overview of the primers design of AAV vector integration assay. The red arrows indicate the location of the primers P1-F and P2-R used for amplifying *P2rx2* V61L loci in mouse genome; P3-F and P4-R for detecting AAV vector; P1-F and ITR-R for detecting AAV vector integration.

(**D**) Quantification of InDel frequency based on the NGS results and AAV vector ITR integration ratio from AAV2-SaCas9-sgRNA-1 edited cells. (**E** to **F**) Gel image (E) and quantification **(**F) of PCR from AAV2-SaCas9-sgRNA-1 edited cochlea and cell line, showing *P2rx2*-ITR integration only in the cell line, not in in vivo hair cells. Red asterisk indicates the *P2rx2*-ITR integration band. (**G**) qPCR analysis of *P2rx2*-ITR RNA level in injected and uninjected cochlea to test if there are P2rx2-ITR transcripts in injected cochlea (n=3). The error bar represents SD.

Figure S3

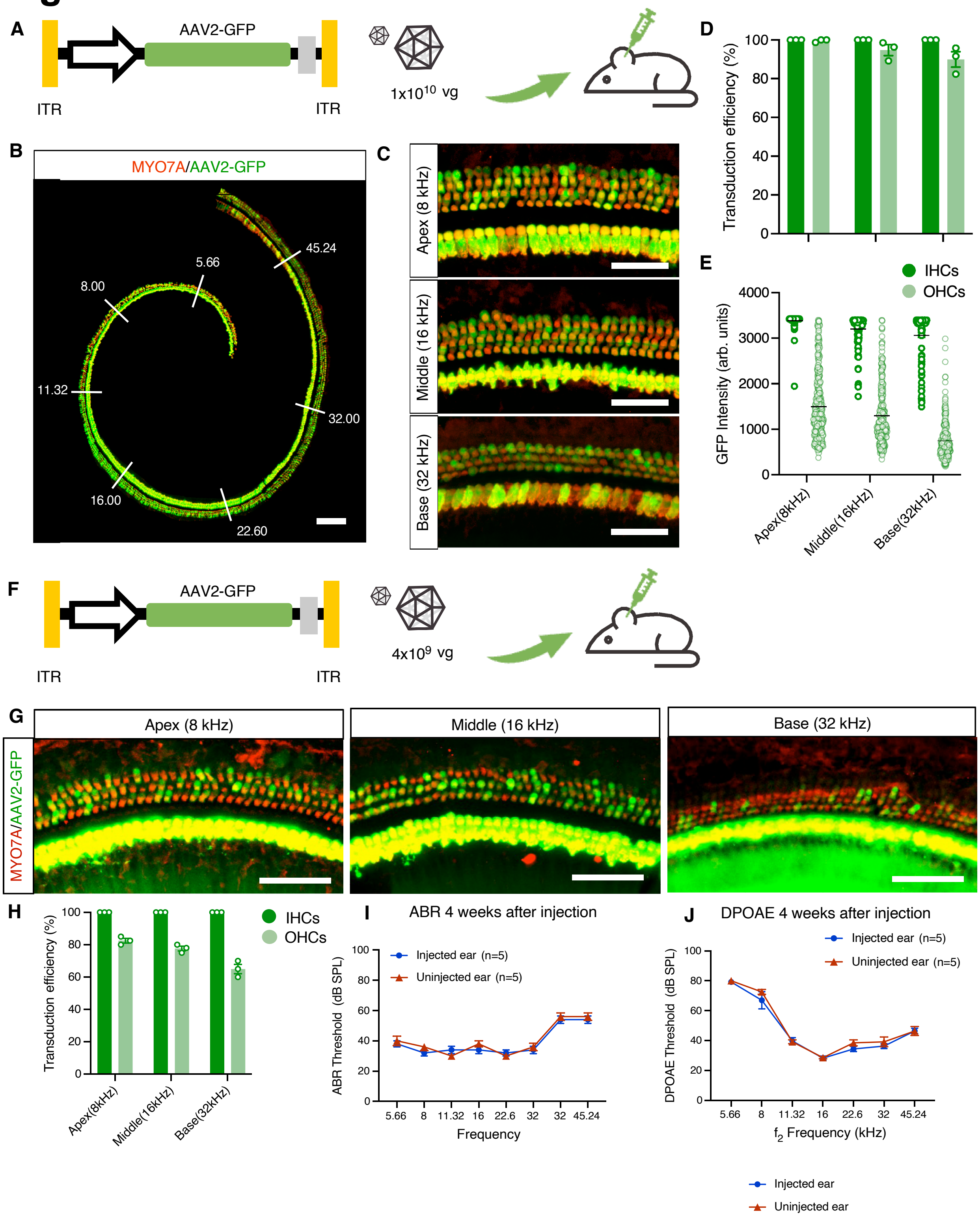

68

69 **Supplemental Figure 3. AAV2-GFP distribution in *P2rx2*$^{V61L/+}$ mouse cochlea by**

RWM injection with canal fenestration. (**A**) Schematic diagram of 1x10^{10} vg AAV2-GFP injection in adult cochlea. (**B**) Whole mount of AAV2-GFP distribution in the cochlear sensorineural epithelium. Scale bar: 200μm. (**C**) Representative distribution of AAV2-GFP in the cochlear apex (8kHz), middle (16kHz) and basal (32kHz) regions at 4 weeks after injection. Scale bar: 50μm**.** (**D**) Quantitation of transduction efficiency of IHCs and OHCs in the apex, middle and basal turn. (**E**) Quantification of GFP fluorescence intensity in transduced hair cells along the cochlear turns; cells measured were from the apical, middle, and basal turns of AAV2-GFP injected cochlea of $P2rx2^{V61L/+}$ mice in IHCs and OHCs. (**F**) Schematic diagram of 4x10^{9} vg AAV2-GFP injection in adult mouse cochlea. (**G**) Representative distribution of AAV2-GFP in the cochlear apex (8kHz), middle (16kHz) and basal (32kHz) regions. (**H**) Quantitation of transduction efficiency of IHCs and OHCs in the apex, middle and basal turn. (**I** to **J**) ABR thresholds (**I**) and DPOAE thresholds at 4 weeks after AAV2-GFP injection between injected ears (blue) and uninjected ears (red). All data are presented as mean ± SEM. Significance was determined by two-way ANOVA test with Bonferroni correction for multiple comparisons. * $p < 0.05$, ** $p < 0.01$, *** $p < 0.001$, **** $p < 0.0001$.

Figure S4

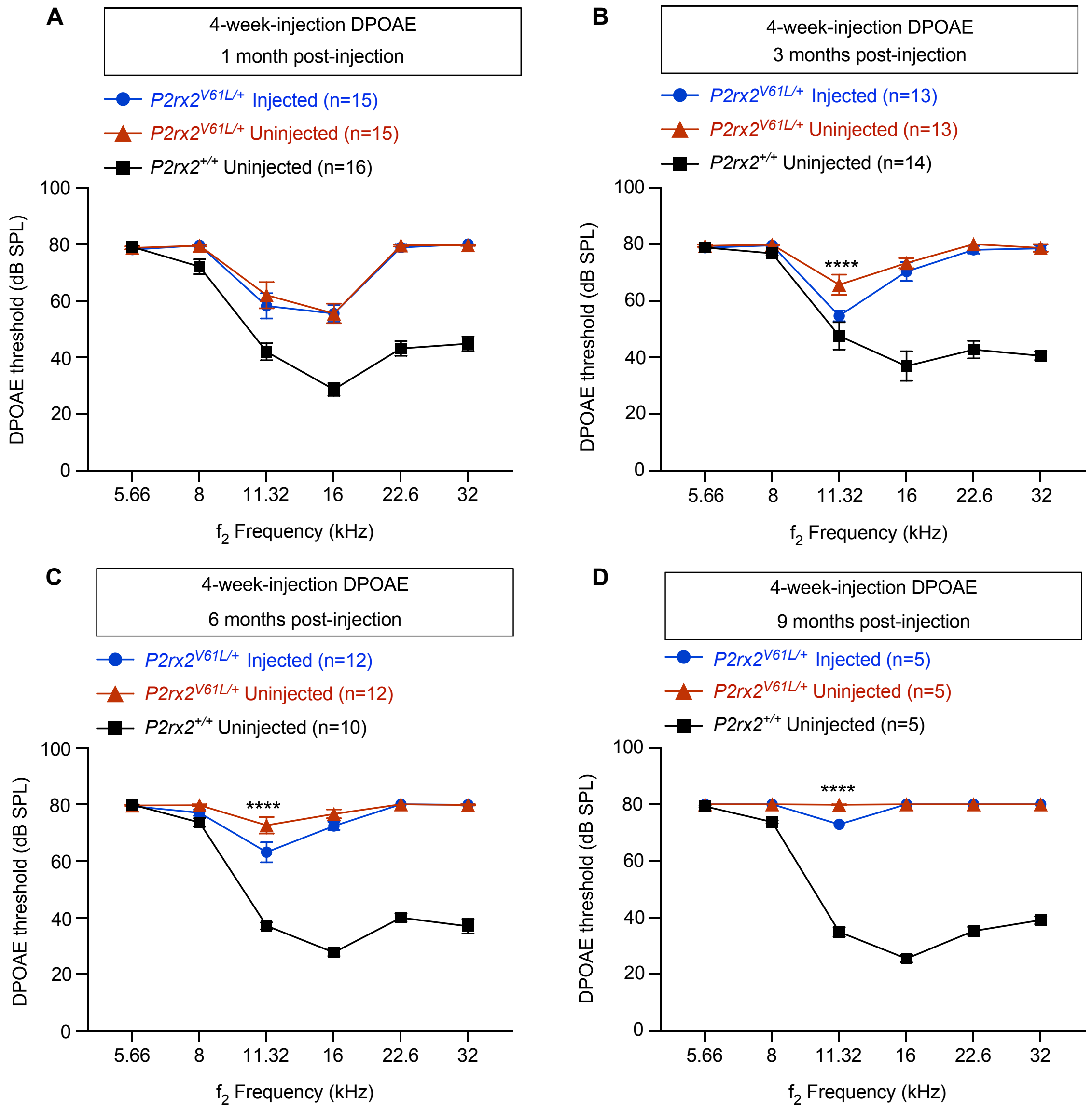

Supplemental Figure 4. 4-week AAV2-SaCas9-sgRNA-1 injection preserves DPOAE in *P2rx2*$^{V61L/+}$ mouse model of DFNA41 long-term. (**A to D**) One month (A), three months (B), six months (C), nine months (D) after injection at 4 weeks of age, the average DPOAE thresholds in injected and uninjected *P2rx2*$^{V61L/+}$, age-matched injected and uninjected WT mice. All data are presented as mean ± SEM. Significance was

determined by two-way ANOVA test with Bonferroni correction for multiple comparisons. * $p < 0.05$, ** $p < 0.01$, *** $p < 0.001$, **** $p < 0.0001$.

Figure S5

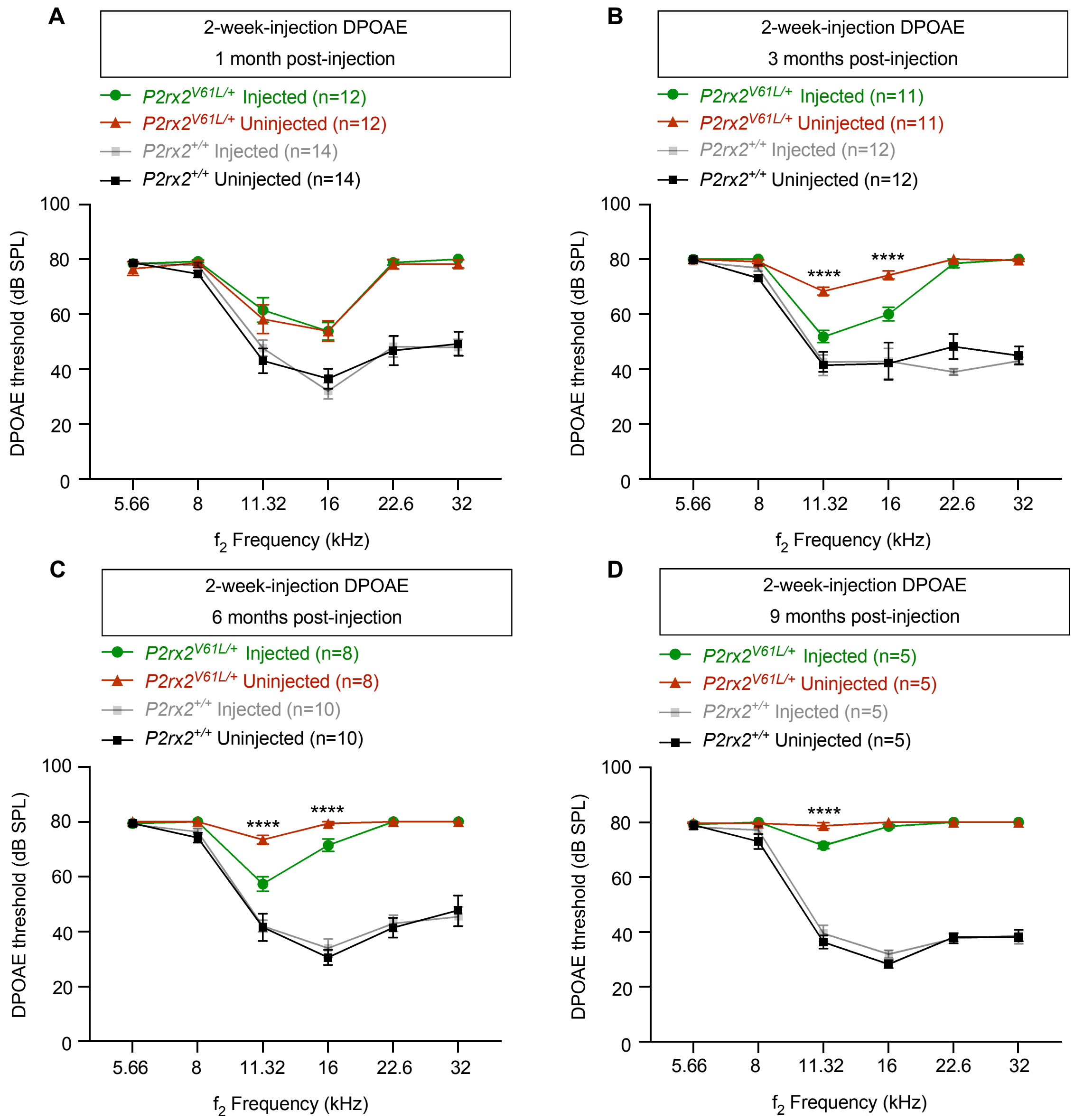

Supplemental Figure 5. 2-week AAV2-SaCas9-sgRNA-1 injection preserves DPOAE in *P2rx2*$^{V61L/+}$ mouse model of DFNA41 long-term. (**A** to **D**) One month (A), three months (B), six months (C), nine months (D) after injection at 2 weeks of age, the average DPOAE thresholds in injected and uninjected *P2rx2*$^{V61L/+}$, age-matched injected and uninjected WT mice. All data are presented as mean ± SEM. Significance was

determined by a two-way ANOVA test with Bonferroni correction for multiple comparisons. * $p < 0.05$, ** $p < 0.01$, *** $p < 0.001$, **** $p < 0.0001$.

Figure S6

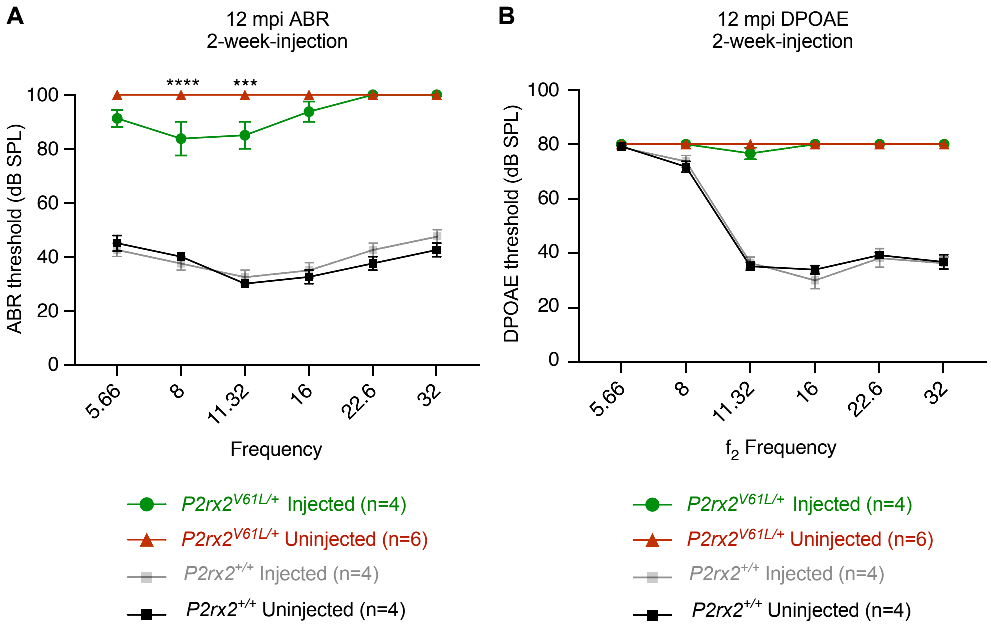

Supplemental Figure 6. Long-term hearing rescue of AAV2-SaCas9-sgRNA-1 in *P2rx2$^{V61L/+}$* mouse model of DFNA41 by early intervention. (**A**) ABR thresholds at 12 months post-2-week injection in injected (green) and uninjected (red) *P2rx2$^{V61L/+}$* ears, age-matched injected and uninjected WT mice (black). (**B**) DPOAE thresholds at 12 months post-2-week injection in injected and uninjected *P2rx2$^{V61L/+}$*ears, age-matched injected and uninjected WT mice. All data are presented as mean ± SEM. Significance was determined by two-way ANOVA test with Bonferroni correction for multiple comparisons. * $p < 0.05$, ** $p < 0.01$, *** $p < 0.001$, **** $p < 0.0001$.

Figure S7

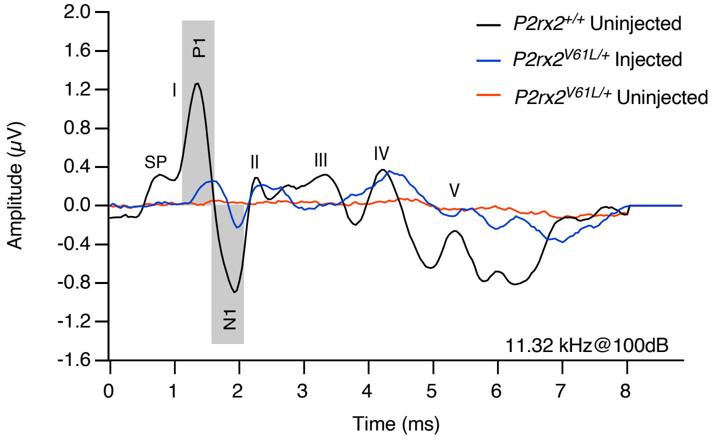

Supplemental Figure 7. Wave 1 amplitude comparison between *$P2rx2^{V61L/+}$* injected and uninfected mice. (**A**) Mean ABR waveform in *$P2rx2^{V61L/+}$* uninjected (red), injected group (blue) and WT (black) at nine months post-injection at 4 weeks old at 100dB 11.32kHz (n=5).

Figure S8

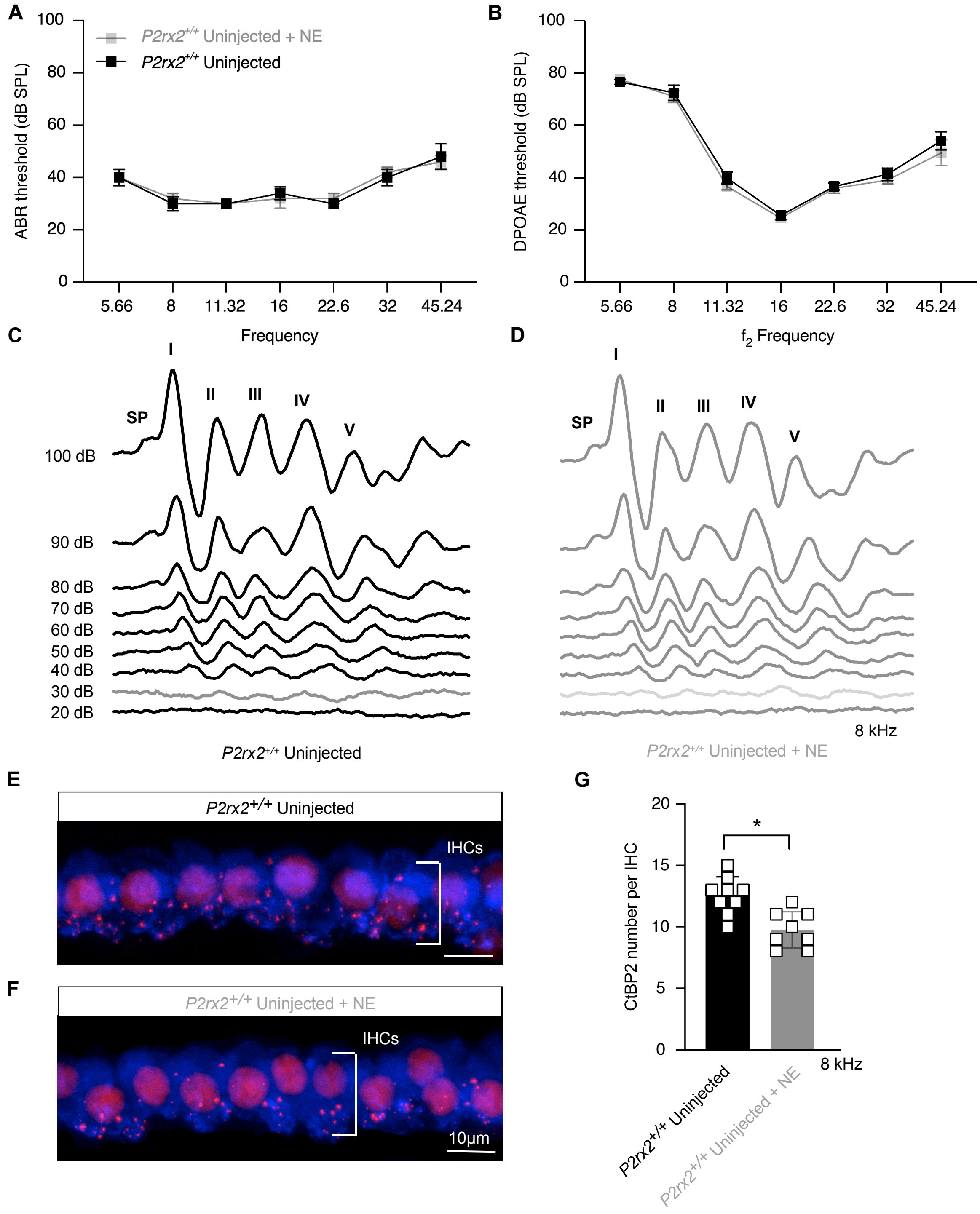

Supplemental Figure 8. Characteristics of *P2rx2*$^{+/+}$ mice in response to noise-induced TTS. (**A** to **B**) ABR thresholds (A) and DPOAE threshold (B) at two weeks post

noise exposure (NE) in uninjected *P2rx2*$^{+/+}$ mice with NE (grey) and uninjected *P2rx2*$^{+/+}$ mice without NE (black). (**C** to **D**) Representative ABR waveforms at 8 kHz of a *P2rx2*$^{+/+}$ mouse without NE (A) and a *P2rx2*$^{+/+}$ mouse following noise exposure (NE) (B). The ABR threshold of 30 dB was detected in *P2rx2*$^{+/+}$ mice without NE (light black) and with NE (light grey), respectively. (**E** to **F**) Immunostaining of pre-ribbon synapses marked by CtBP2 (red) of without NE (C) and with NE (D) *P2rx2*$^{+/+}$ mice followed by noise exposure. MYO7A labels HC (blue). Scale bars: 10µm. (**G**) Statistical analysis of Ctbp2 number per IHC in WT mice with NE and without NE group. All data are presented as mean ± SEM. Significance was determined by two-way ANOVA test with Bonferroni correction for multiple comparisons. * $p < 0.05$, ** $p < 0.01$, *** $p < 0.001$, **** $p < 0.0001$.

Figure S9

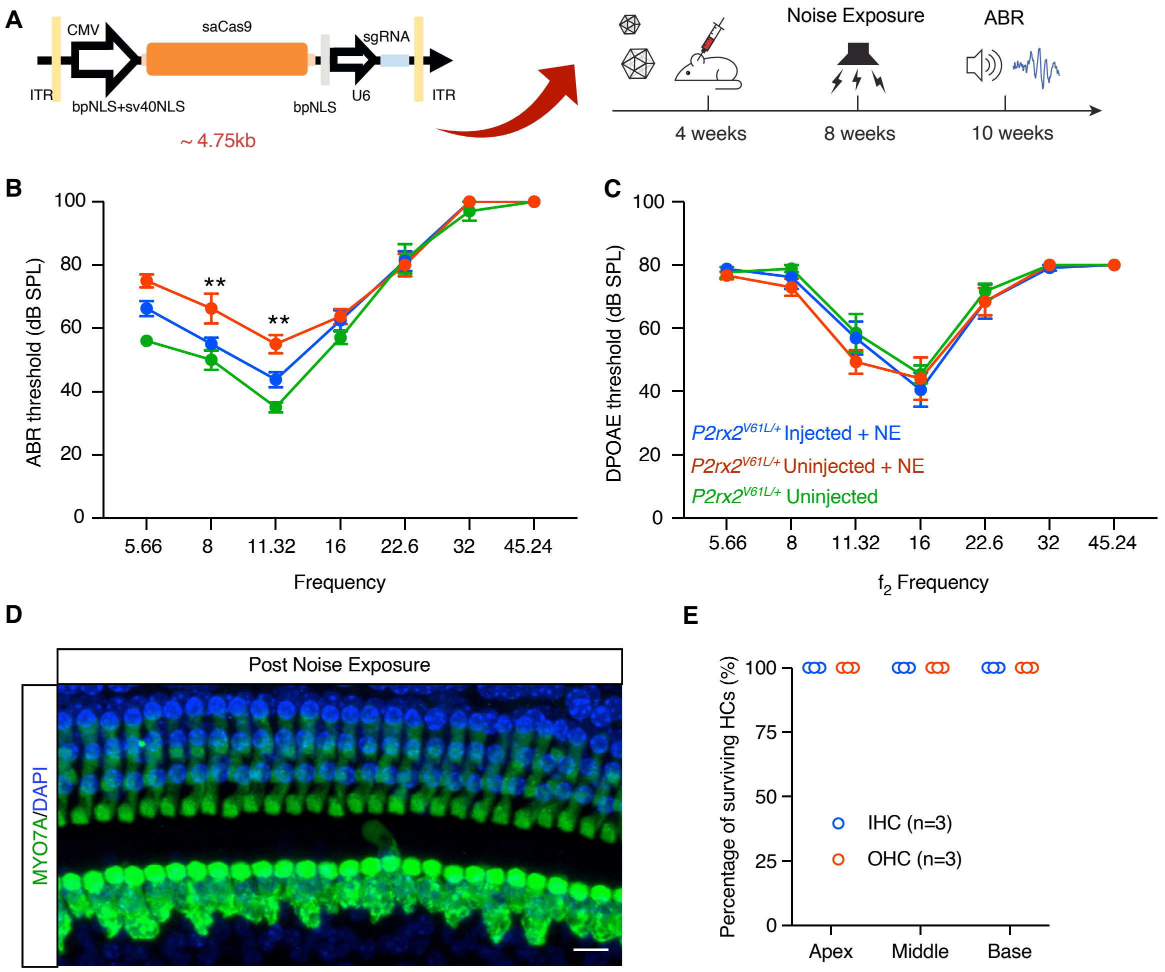

Supplemental Figure 9. AAV2-SaCas9-sgRNA-1 injection partially attenuates increased sensitivity to noise-induced hearing loss (NIHL) in *P2rx2$^{V61L/+}$* mouse model of DFNA41 at 4-week-old intervention. (**A**) Schematic representation of the experimental design. (**B** to **C**) ABR thresholds (B) and DPAOE threshold (C) among different groups: injected and noise exposed *P2rx2$^{V61L/+}$* (blue), uninjected and noise exposed *P2rx2$^{V61L/+}$* (red), and uninjected *P2rx2$^{V61L/+}$* without noise exposure (green). (**D**) MYO7A (Green) and DAPI (Blue) immunolabeled HCs in *P2rx2$^{V61L/+}$* mouse 2 weeks after noise exposure. (**E**) Statistical analysis of IHCs and OHCs surviving percentage. All data are presented as mean ± SEM. Significance was determined by two-way

ANOVA test with Bonferroni correction for multiple comparisons. * $p < 0.05$, ** $p < 0.01$, *** $p < 0.001$, **** $p < 0.0001$.

Figure S10

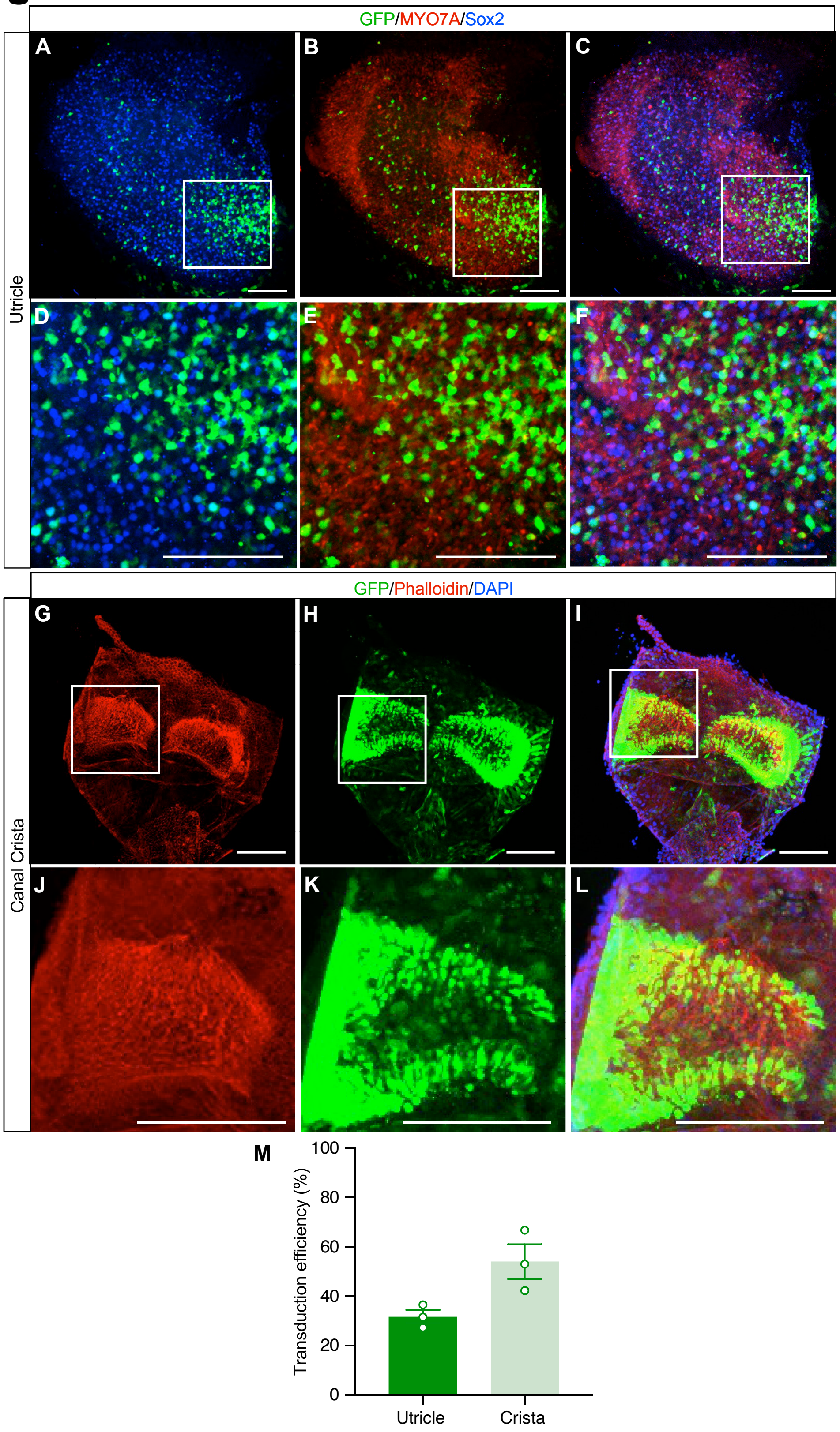

181

Supplemental Figure 10. AAV2-GFP distribution in the utricle of *P2rx2*$^{V61L/+}$ mouse model. (**A** to **C**) Overviews of AAV2-GFP distribution in the vestibular utricle under low magnification labeled with GFP (green), MYO7A (red) and Sox2 (blue). (**E** to **F**) Higher magnification of represented images shows the distribution of AAV2-GFP in the utricle (D- enlarged inset in A; E- enlarged inset in B; F- enlarged inset in C). (**G** to **H**) Overviews of AAV2-GFP distribution in the canal crista labeled with GFP (green), Phalloidin (red) and DAPI (blue) under low magnification. (**J** to **L**) Higher magnification of represented images shows the distribution of AAV2-GFP in the crista (J- enlarged inset in G; K- enlarged inset in H; L- enlarged inset in I). (**G**) Quantitation of transduction efficiency in utricle and crista in percentage. Scale bar: 100μm.

AAV-CMV-SaCas9-sgRNA-1:

CCTGCAGGCAGCTGCGCGCTCGCTCGCTCACTGAGGCCGCCCGGGCGTCGGGCGACCTTTGGTCGCCCGGCCTCAGTGAGC
GAGCGAGCGCGCAGAGAGGGAGTGGCCAACTCCATCACTAGGGGTTCCTGCGGCCTCTAGACTCGAGGCGTTGACATTGATT
ATTGACTAGTTATTAATAGTAATCAATTACGGGGTCATTAGTTCATAGCCCATATATGGAGTTCCGCGTTACATAACTTACGGTAA
ATGGCCCGCCTGGCTGACCGCCCAACGACCCCCGCCCATTGACGTCAATAATGACGTATGTTCCCATAGTAACGCCAATAGGG
ACTTTCCATTGACGTCAATGGGTGGAGTATTTACGGTAAACTGCCCACTTGGCAGTACATCAAGTGTATCATATGCCAAGTACG
CCCCCTATTGACGTCAATGACGGTAAATGGCCCGCCTGGCATTATGCCCAGTACATGACCTTATGGGACTTTCCTACTTGGCAG
TACATCTACGTATTAGTCATCGCTATTACCATGGTGATGCGGTTTTGGCAGTACATCAATGGGCGTGGATAGCGGTTTGACTCA
CGGGGATTTCCAAGTCTCCACCCCATTGACGTCAATGGGAGTTTGTTTTGGCACCAAAATCAACGGGACTTTCCAAAATGTCGT
AACAACTCCGCCCCATTGACGCAAATGGGCGGTAGGCGTGTACGGTGGGAGGTCTATATAAGCAGAGCTCTCTGGCTAACTAG
CCACCATGGGCAAACGCCCAGCAGCTACAAAGAAGGCAGGTCAAGCCAAGAAAAAGAAAggaGCCCCAAAGAAGAAGCGGAAG
GTCGGTtccAAGCGGAACTACATCCTGGGCCTGGACATCGGCATCACCAGCGTGGGCTACGGCATCATCGACTACGAGACACG
GGACGTGATCGATGCCGGCGTGCGGCTGTTCAAAGAGGCCAACGTGGAAAACAACGAGGGCAGGCGGAGCAAGAGAGGCGC
CAGAAGGCTGAAGCGGCGGAGGCGGCATAGAATCCAGAGAGTGAAGAAGCTGCTGTTCGACTACAACCTGCTGACCGACCAC
AGCGAGCTGAGCGGCATCAACCCCTACGAGGCCAGAGTGAAGGGCCTGAGCCAGAAGCTGAGCGAGGAAGAGTTCTCTGCC
GCCCTGCTGCACCTGGCCAAGAGAAGAGGCGTGCACAACGTGAACGAGGTGGAAGAGGACACCGGCAACGAGCTGTCCACC
AAAGAGCAGATCAGCCGGAACAGCAAGGCCCTGGAAGAGAAATACGTGGCCGAACTGCAGCTGGAACGGCTGAAGAAAGACG
GCGAAGTGCGGGGCAGCATCAACAGATTCAAGACCAGCGACTACGTGAAAGAAGCCAAACAGCTGCTGAAGGTGCAGAAGGC
CTACCACCAGCTGGACCAGAGCTTCATCGACACCTACATCGACCTGCTGGAAACCCGGCGGACCTACTATGAGGGACCTGGC
GAGGGCAGCCCCTTCGGCTGGAAGGACATCAAAGAATGGTACGAGATGCTGATGGGCCACTGCACCTACTTCCCCGAGGAAC
TGCGGAGCGTGAAGTACGCCTACAACGCCGACCTGTACAACGCCCTGAACGACCTGAACAATCTCGTGATCACCAGGGACGA
GAACGAGAAGCTGGAATATTACGAGAAGTTCCAGATCATCGAGAACGTGTTCAAGCAGAAGAAGAAGCCCACCCTGAAGCAGA
TCGCCAAAGAAATCCTCGTGAACGAAGAGGATATTAAGGGCTACAGAGTGACCAGCACCGGCAAGCCCGAGTTCACCAACCTG
AAGGTGTACCACGACATCAAGGACATTACCGCCCGGAAAGAGATTATTGAGAACGCCGAGCTGCTGGATCAGATTGCCAAGAT
CCTGACCATCTACCAGAGCAGCGAGGACATCCAGGAAGAACTGACCAATCTGAACTCCGAGCTGACCCAGGAAGAGATCGAG
CAGATCTCTAATCTGAAGGGCTATACCGGCACCCACAACCTGAGCCTGAAGGCCATCAACCTGATCCTGGACGAGCTGTGGCA
CACCAACGACAACCAGATCGCTATCTTCAACCGGCTGAAGCTGGTGCCCAAGAAGGTGGACCTGTCCCAGCAGAAAGAGATCC
CCACCACCCTGGTGGACGACTTCATCCTGAGCCCCGTCGTGAAGAGAAGCTTCATCCAGAGCATCAAAGTGATCAACGCCATC
ATCAAGAAGTACGGCCTGCCCAACGACATCATTATCGAGCTGGCCCGCGAGAAGAACTCCAAGGACGCCCAGAAAATGATCAA
CGAGATGCAGAAGCGGAACCGGCAGACCAACGAGCGGATCGAGGAAATCATCCGGACCACCGGCAAAGAGAACGCCAAGTAC
CTGATCGAGAAGATCAAGCTGCACGACATGCAGGAAGGCAAGTGCCTGTACAGCCTGGAAGCCATCCCTCTGGAAGATCTGCT
GAACAACCCCTTCAACTATGAGGTGGACCACATCATCCCCAGAAGCGTGTCCTTCGACAACAGCTTCAACAACAAGGTGCTCGT
GAAGCAGGAAGAAAACAGCAAGAAGGGCAACCGGACCCCATTCCAGTACCTGAGCAGCAGCGACAGCAAGATCAGCTACGAA
ACCTTCAAGAAGCACATCCTGAATCTGGCCAAGGGCAAGGGCAGAATCAGCAAGACCAAGAAAGAGTATCTGCTGGAAGAACG
GGACATCAACAGGTTCTCCGTGCAGAAAGACTTCATCAACCGGAACCTGGTGGATACCAGATACGCCACCAGAGGCCTGATGA
ACCTGCTGCGGAGCTACTTCAGAGTGAACAACCTGGACGTGAAAGTGAAGTCCATCAATGGCGGCTTCACCAGCTTTCTGCGG
CGGAAGTGGAAGTTTAAGAAAGAGCGGAACAAGGGGTACAAGCACCACGCCGAGGACGCCCTGATCATTGCCAACGCCGATT
TCATCTTCAAAGAGTGGAAGAAACTGGACAAGGCCAAAAAAGTGATGGAAAACCAGATGTTCGAGGAAAAGCAGGCCGAGAGC
ATGCCCGAGATCGAAACCGAGCAGGAGTACAAAGAGATCTTCATCACCCCCCACCAGATCAAGCACATTAAGGACTTCAAGGA
CTACAAGTACAGCCACCGGGTGGACAAGAAGCCTAATAGAGAGCTGATTAACGACACCCTGTACTCCACCCGGAAGGACGACA
AGGGCAACACCCTGATCGTGAACAATCTGAACGGCCTGTACGACAAGGACAATGACAAGCTGAAAAAGCTGATCAACAAGAGC
CCCGAAAAGCTGCTGATGTACCACCACGACCCCCAGACCTACCAGAAACTGAAGCTGATTATGGAACAGTACGGCGACGAGAA
GAATCCCCTGTACAAGTACTACGAGGAAACCGGGAACTACCTGACCAAGTACTCCAAAAAGGACAACGGCCCCGTGATCAAGA
AGATTAAGTATTACGGCAACAAACTGAACGCCCATCTGGACATCACCGACGACTACCCCAACAGCAGAAACAAGGTCGTGAAG
CTGTCCCTGAAGCCCTACAGATTCGACGTGTACCTGGACAATGGCGTGTACAAGTTCGTGACCGTGAAGAATCTGGATGTGAT
CAAAAAAGAAAACTACTACGAAGTGAATAGCAAGTGCTATGAGGAAGCTAAGAAGCTGAAGAAGATCAGCAACCAGGCCGAGTT
TATCGCCTCCTTCTACAACAACGATCTGATCAAGATCAACGGCGAGCTGTATAGAGTGATCGGCGTGAACAACGACCTGCTGAA
CCGGATCGAAGTGAACATGATCGACATCACCTACCGCGAGTACCTGGAAAACATGAACGACAAGAGGCCCCCCAGGATCATTA
AGACAATCGCCTCCAAGACCCAGAGCATTAAGAAGTACAGCACAGACATTCTGGGCAACCTGTATGAAGTGAAATCTAAGAAGC
ACCCTCAGATCATCAAAAAGGGCAAAAGGCCGGCGGCCACGAAAAAGGCCGGCCAGGCAAAAAAGAAAAAGGGATCCTACCC
ATACGATGTTCCAGATTACGCTTAAGAATTCGCTGATCAGCCTCGACTGTGCCTTCTAGTTGCCAGCCATCTGTTGTTTGCCCCT
CCCCCGTGCCTTCCTTGACCCTGGAAGGTGCCACTCCCACTGTCCTTTCCTAATAAAATGAGGAAATTGCATCGCATTGTCTGA
GTAGGTGTCATTCTATTCTGGGGGGTGGGGTGGGGCAGGACAGCAAGGGGGAGGATTGGGAAGAGAATAGCAGGCATGCTG
GGGAGGTACCGAGGGCCTATTTCCCATGATTCCTTCATATTTGCATATACGATACAAGGCTGTTAGAGAGATAATTGGAATTAAT
TTGACTGTAAACACAAAGATATTAGTACAAAATACGTGACGTAGAAAGTAATAATTTCTTGGGTAGTTTGCAGTTTTAAAATTATG
TTTTAAAATGGACTATCATATGCTTACCGTAACTTGAAAGTATTTCGATTTCTTGGCTTTATATATCTTGTGGAAAGGACGAAACA
CCGcacgatgaagaGgtacctggttAtagtactctgtaatgaaaattacagaatctactaTaacaaggcaaaatgccgtgtttatctcgtcaacttgttggcgagaTTTTTTTGCGG
CCGCAGGAACCCCTAGTGATGGAGTTGGCCACTCCCTCTCTGCGCGCTCGCTCGCTCACTGAGGCCGGGCGACCAAAGGTCG
CCCGACGCCCGGGCTTTGCCCGGGCGGCCTCAGTGAGCGAGCGAGCGCGCAGCTGCCTGCAGG

ITR, CMV Promoter, NLSs, SaCas9-KKH coding sequence, HA Tag, PloyA, U6 Promoter, sgRNA

Supplementary Fig. 1 | DNA sequence of AAV-CMV-SaCas9-sgRNA-1.

Name	Sequence (5'-3')
P1	TTCTGCTTTACTTCGTGTGGTGCG
P2	TTTGTGTTCCGACATGGTGATCCC
P3	GCTTCAACAACAAGGTGCTCGTG
P4	CTGTTGATGTCCCGTTCTTCCAG
AAV ITR	GGAACCCCTAGTGATGGAGTT
P2rx2-NGS-F	TTCTGCTTTACTTCGTGTGGTGCG
P2rx2-NGS-R	TTTGTGTTCCGACATGGTGATCCC
hu-P2rx2-NGS-F	CGTGCCTGCGGGCGGGACTCAG
hu-P2rx2-NGS-R	CGCCCACTAGGGTAGGGTGCACG
hu-P2rx2-NGS-R	CGCCCACTAGGGTAGGGTGCACG
hu-P2rx2-NGS-R	CGCCCACTAGGGTAGGGTGCACG

Supplementary Table. 1 | Primers used in this study.

Primer	Sequence (5'-3')	Primer	Sequence (5'-3')
OT1-F	ATCTGGTATGCTGCCCACAAAGAG	OT1-R	CAGTTTTCTGTGGAGAAAGCAGAG
OT2-F	CAGTAGCAATTGGACCAGCTTCTG	OT2-R	AATGCTATACACAGTCACACACTAG
OT3-F	CACCCATCAGTGTGGTCCAGTGG	OT3-R	CTATGGCCGCTGATGTGATGCCAG
OT4-F	GGAAAGAAATGTTTATGCTTTGGAAG	OT4-R	GGACCATGCTTTTGTAGCAAAGTG
OT5-F	CTGGTCTGGTGCCCTTTCCAAG	OT5-R	TATATTCTCCTGAAAGGAAGTAAG
OT6-F	GCAGGAGGGAGATTTGTCAAAGAG	OT6-R	CCATCTCAAAAACAAACAACTGTGG
OT7-F	GAAATGGAAATCTATACAGAGGATG	OT7-R	AAAGCACCAGTTTCAGCTGATGGG

Supplementary Table. 2 | Primers used for offtarget analysis in this study.

Name	Sequence (5'-3')
sgRNA-1	GCACGATGAAGAGGTACCTG TGGGAT
sgRNA-2	GCACGATGAAGAGGTACCTG TGG
sgRNA-3	CACGATGAAGAGGTACCTGT GGG
sgRNA-4	TGAAGAGGTACCTGTGGGAT AGG
sgRNA-5	GAAGAGGTACCTGTGGGATA GGG
sghuP2rx2	GGTAGCTTTTCTGCACGATGA ATAAGT

Supplementary Table. 3 | sgRNA protospacer sequences used in this study. Red fonts indicate the PAM sequence.

Supplementary Methods

Isolation and culture of Primary Fibroblast from Mice

P2rx2$^{V61L/+}$ and WT mice were euthanized and cleaned with 70% ethanol. The dorsal skin (approximately 1 cm diameter) of the mice was collected and rinsed with DPBS; subcutaneous fat was removed by forceps. Subsequently, the samples were cut into small fragments and incubated with Dispase II (Sigma-Aldrich, USA) for overnight at 4 °C. The dermal layers of the skin were separated and further subjected to incubation with type I collagenase (1 mg/ml Gibco, USA) for 2 hours at 37 degrees. The resulting cell suspension was strained using a 40-micron strainer and centrifuged at 950 rpm to obtain the cell pellet. The cell pellet was seeded in T-75 flask containing DMEM high glucose media (Gibco, USA) containing 10 % FBS (Gibco, USA). Fibroblasts were cultured for about 2–3 days to reach approximately90% confluence, then passaged in T75 flasks with TrypLE Express and cultured in DMEM: F12 medium (ThermoFisher) with 10% fetal bovine serum (FBS) supplemented with GlutaMax (ThermoFisher).

Construction of *P2rx2* V61L cell line using PiggyBac

Mouse *P2rx2* V61L frgment harboring the +14C>A mutation was amplified by PCR from *P2rx2*$^{V61L/V61L}$ mouse genomic DNA and cloned into the PiggyBac donor backbone (PB-CAG-mNeonGreen-P2A-BSD-polyA) using Gibson Assembly. The PB donor plasmid was co-transfected with PiggyBac transposon vector (PB210PA, System Biosciences) into HEI-OC1 cells (RRID: CVCL_D899). Cells were cultured and selected in the medium containing 10 μg/mL Blasticidin for 2 weeks. Human *P2rx2 V60L* fragment, mutation was cloned into the same PiggyBac donor backbone and transtracted into

HEK-293T cells (CRL-11268). PCR and sequencing analysis confirmed successful insertion.

RNA isolation and qRT-PCR

Total RNA was extracted from inner ear tissue using the ReliaPrep RNA Tissue Miniprep System (Promega, z6111). Then first-strand cDNA was produced using ProtoScript® II First Strand cDNA Synthesis Kit (NEB, E6560s). Real time quantitative PCR was performed using Power SYBR Green PCR Master Mix (Applied Biosystems, 4368708) on the ABI QuantStudio 3 Flex Real-Time PCR System (Applied Biosystems).

AAV vector integration assay

HEK 293T-P2rx2-V61L cells were treated with AAV2-CMV-SaCas9-sgRNA-1 of different dosages from 1 to 10^5 genomic copies per cell. Cells were collected 7 days later, and genomic DNA was isolated. Primers P1-F/ITR-R were used for detecting AAV vector integration. For in vivo integration detection, Primers P1-F/P2-R and primers P1-F/ITR-R were used to amply genomic fragments from isolated hair cells, then PCR products were merged for NGS analysis. Related primers were listed in Supplementary Table. 1

Off-target analysis

To identify off-target sites, the CIRCLE-seq was performed essentially as previously described with minor modifications. Briefly, 50 µg genomic DNA was purified from HEI-OC1 cells. Genomic DNA was sheared using Tn5 and circularized. In vitro cleavage

reaction of circularized genomic DNA is performed with SaCas9 protein (synthego)/sgRNA-1. Then the sequencing libraries were prepared and sequenced on an Illumina Miseq. Off-target sites were identified with the standard pipeline. The negative control sample were treated with the Cas9 alone to assess background. Besides, we listed all 7 potential off-target sites identified by the cutting frequency determination (CFD) score (1). We designed PCR primers on the two flanks of each sgRNA target sequences for amplifying 250-280 bp DNA fragments. Then amplified fragments were purified for NGS to identify whether there is any off-target mutation. Primers were listed in Supplementary Table. 2.

Scanning electron microscopy

Following euthanasia, the temporal bones of mice were carefully separated from the surrounding skull base. Small holes were created in the round window and oval window membranes to ensure optimal fixation, and a small apical piece of bone near the helicotrema was removed. The cochleae were then immersed in a solution of 2.5% glutaraldehyde in 0.1 M sodium cacodylate buffer supplemented with 20 mM $CaCl_2$ for 60 minutes room temperature or overnight at 4 degrees. After fixation, the cochleae were washed with distilled water and subjected to decalcification for 24-48 hours using 120 mM EDTA at pH 7.4. Subsequently, the cochleae were rinsed again in distilled water, and sections of the organ of Corti were carefully micro dissected from the surrounding supporting tissue and bone. To prepare the tissues for analysis, a gradual ethanol series from 50% to 100% was used for dehydration. The dehydrated tissues were placed in a critical point dryer (Tousimis 931) and mounted on double-sided conductive carbon tape. A thin layer of platinum (approximately 5 nm) was coated onto

the tissues using a Leica ACE600 system. Finally, the samples were examined using a Hitachi S-4700 scanning electron microscope, utilizing a 5.0-kV accelerating voltage, in the Harvard Medical School Imaging and Analysis Core to capture the images.

Reference

1. Doench JG, et al. Optimized sgRNA design to maximize activity and minimize off-target effects of CRISPR-Cas9. *Nat Biotechnol.* 2016;34(2):184-91.
